# Improving living and dying for people with advanced dementia living in care homes: a realist review of Namaste Care and other multisensory interventions

**DOI:** 10.1186/s12877-018-0995-9

**Published:** 2018-12-06

**Authors:** Frances Bunn, Jennifer Lynch, Claire Goodman, Rachel Sharpe, Catherine Walshe, Nancy Preston, Katherine Froggatt

**Affiliations:** 1 Centre for Research in Primary and Community Care, University of Hertfordshire, College Lane, Hatfield, Hertfordshire, AL10 9AB UK; 2 International Observatory on End of Life Care, Faculty of Health and Medicine, Lancaster University, Lancaster, LA1 4YG UK

**Keywords:** Realist review, Realist synthesis, Dementia, Namaste care, Multisensory, Palliative care

## Abstract

**Background:**

Seventy percent of people with advanced dementia live and die in care homes. Multisensory approaches, such as Namaste Care, have been developed to improve the quality of life and dying for people with advanced dementia but little is known about effectiveness or optimum delivery. The aim of this review was to develop an explanatory account of how the Namaste Care intervention might work, on what outcomes, and in what circumstances.

**Methods:**

This is a realist review involving scoping of the literature and stakeholder interviews to develop theoretical explanations of how interventions might work, systematic searches of the evidence to test and develop the theories, and their validation with a purposive sample of stakeholders. Twenty stakeholders - user/patient representatives, dementia care providers, care home staff, researchers -took part in interviews and/or workshops.

**Results:**

We included 85 papers. Eight focused on Namaste Care and the remainder on other types of sensory interventions such as music therapy or massage. We identified three context-mechanism-outcome configurations which together provide an explanatory account of what needs to be in place for Namaste Care to work for people living with advanced dementia. This includes: providing structured access to social and physical stimulation, equipping care home staff to cope effectively with complex behaviours and variable responses, and providing a framework for person-centred care. A key overarching theme concerned the importance of activities that enabled the development of moments of connection for people with advanced dementia.

**Conclusions:**

This realist review provides a coherent account of how Namaste Care, and other multisensory interventions might work. It provides practitioners and researchers with a framework to judge the feasibility and likely success of Namaste Care in long term settings. Key for staff and residents is that the intervention triggers feelings of familiarity, reassurance, engagement and connection.

**Study registration:**

This study is registered as PROSPERO CRD42016047512

**Electronic supplementary material:**

The online version of this article (10.1186/s12877-018-0995-9) contains supplementary material, which is available to authorized users.

## Background

Approximately one-third of people over the age of 65 will die with or from dementia and, in the United Kingdom, the majority with advanced dementia will die in care homes [1–3]. Dementia is a terminal neurodegenerative disease and individuals with advanced dementia are likely to have complex physical and psychiatric needs [4, 5]. There is a lack of evidence-based therapeutic interventions for people with advanced dementia. A Cochrane systematic review on palliative care interventions in advanced dementia found only two studies that met their inclusion criteria [6].

Interventions focused on improving end of life care for people with advanced dementia are being developed and tested for use with care home residents [7]. One such intervention is Namaste Care. Developed in the United States Namaste Care aims to address the physical, sensory and emotional needs of people with advanced dementia [8]. It is designed to be delivered twice a day, seven days a week, and as a group intervention whereby residents receive one-to-one attention in a communal space dedicated to Namaste Care [9]. The intervention is focused on sensory stimulation and includes personalized activities to reflect the preferences of individuals [8]. The intervention aims to increase the amount of time staff spend with residents, reduce isolation in people with advanced dementia and improve the quality of living and dying at the end of life. Staff are trained to deliver the programme [9].

There is evidence that Namaste Care can increase social interaction, reduce the severity of behavioural and physical symptoms such as agitation [8, 10, 11] and, potentially, lead to cost savings via a reduction in the use of psychotropic medication [12, 13]. Qualitative evidence also suggests it can increase family and staff satisfaction with care [8]. However, these findings are from non-randomised studies, none of which have compared Namaste Care with other approaches to palliative and end of life care for this population. We do not currently know how best to deliver this intervention or whether benefits can be demonstrated for people with advanced dementia.

Namaste Care is a complex multicomponent intervention. Realist review is a systematic, theory-driven approach that aims to make explicit the underlying processes, structures or reasoning (mechanisms) of how and why complex interventions work (or not) in particular settings or contexts [14–16]. The aim of this review was to understand how the Namaste Care intervention might achieve particular outcomes, and in what circumstances. The focus was on the contextual conditions and mechanisms that influence how end of life care for people with advanced dementia is effectively managed in care homes.

## Methods

We conducted a stakeholder driven, iterative, two phase realist review. The approach draws on the work of Pawson [17, 18] and is informed by RAMESES guidance on the conduct and reporting of realist reviews [15]. Realist review takes account of a broad and eclectic evidence base, including experiential knowledge [15, 16, 19]. The purpose of this review was to develop an explanatory account or programme theory about Namaste Care and how it might work for people with advanced dementia living in long-term care settings. Programme theory comprise configurations of context (the background conditions in which interventions are delivered and in which mechanisms are triggered), mechanism (the responses or changes that are brought about through a programme within a particular context) and outcomes. The development of these context-mechanism-outcome (CMO) configurations is iterative involving data collection, theorising and stakeholder engagement. Stakeholders with direct experience of providing end of life care to people with dementia were involved in defining the scope of the review and later in validating the programme theory.

### Phase 1: Defining the scope of the realist review – Concept mining and theory development

To develop our initial programme theory we searched for all available literature describing the implementation or use of Namaste Care. This involved searches in PubMed and CINAHL using the free text term Namaste, and lateral searches of reference lists and a book by Joyce Simard the originator of Namaste Care [9]. We included research studies of any design and descriptive items in non-academic journals. In addition, we conducted face-to-face or telephone interviews with 11 participants involved in delivering Namaste Care, training care home staff in Namaste Care, and researching dementia and/or end of life care. Participants were based in the UK, The Netherlands and the USA. Participants were recruited for their known expertise and through snowball sampling. Interviews were conducted using realist principles [20] and were guided by a topic guide (see Additional file 1). The purpose of the stakeholder consultation at this stage was to explore assumptions about Namaste Care, including what were considered essential components of the intervention, how it was thought to work and on what outcomes. Research Ethics Committee approval was obtained from Lancaster University 17/wa/0378.

Findings from the literature and interviews were used to develop preliminary theory in the form of 13 explanatory ‘if-then’ statements (see Additional file 2). If-Then’ statements are the identification of an intervention/activity linked to outcome(s). They contain references to contexts and mechanisms although these may not be very explicit at this stage [21]. Following this, we organised a workshop to review and refine the theory. Participants were chosen based on expertise in Namaste Care and/or dementia or end of life care. The workshop included seven external participants (three of whom had participated in interviews), and six members of the study team (one of whom is a Participant and Public Perspective lead). At the workshop members of the project team presented the preliminary findings from the scoping, the outcomes identified from the literature, and the if-then statements. We adapted nominal group technique to facilitate discussion of the if-then statements. Nominal group technique is a process that promotes the generation of ideas to develop a set of priorities ideas and enables the participation of all group members [22]. Participants comments were recorded, and statements ranked by participants in order of importance. After the workshop members of the project team who had attended the workshop reviewed the if-then statements, and the rankings, and grouped them into three categories:

1. How Namaste Care is introduced to the care home, including structure of the intervention, frequency and resources
2. Characteristics/approach of the care home staff and characteristics of the Namaste Care programme, for example staff providing person-centred care and engaging in biography work with residents
3. How Namaste Care is delivered, including meaningful activities involving all five senses and adaptation of activities to individual circumstances and preferences

These categories became the basis for three preliminary CMOs (see Additional file 2 for details) which were taken forward for testing in Phase 2.

### Phase 2: Retrieval, review and synthesis

#### Inclusion criteria and study identification

In Phase 2 we undertook systematic searches to test and develop the three CMOs identified in Phase 1. In Phase 1 our searches had focused on the Namaste Care Literature. In Phase 2 we broadened the searches to include studies that drew on similar principles or approaches to Namaste Care. The rationale for this was that they offered opportunities for transferable learning and allowed us to test aspects of our programme theory or the mechanisms of action. The inclusion criteria for studies were as follows:

- All or some participants with advanced dementia. This includes studies where it was based on authors reports and those that provided more formal definitions or used scores such as the Mini Mental State Examination (MMSE).
- People living in long-term care institution (e.g. a care home or nursing home)
- Interventions that drew on similar principles to Namaste Care or included components of Namaste Care identified in Phase 1 (e.g. music therapy, massage, aromatherapy). This included group based or one-to-one interventions. Interventions could be delivered by care home staff or external facilitators.
- Published and unpublished studies of any design

The searches focused on papers published in the last 10 years to reflect the rapid expansion of work and interest in the research area. We searched PubMed, Scopus and CINAHL. Search terms and dates are given in Table 1. In addition, we undertook lateral searching such as forward and backward citation tracking. The purpose of the searches was to identify sufficient evidence for building and testing our programme theory [23]. The test for conceptual saturation was applied iteratively through regular discussion among team members involved in data extraction (FB, JL, RS, CG). [14].

**Table 1:** Search terms used in PubMed in Phase 2 (search terms were adapted as appropriate for other databases)

PubMed search 1 (run 24.4.17, focused on elements of Namaste Care intervention such as massage, music, sensory stimulation)	
sensory[Title/Abstract] OR touch[Title/Abstract] OR senses[Title/Abstract] OR massage[Title/Abstract] OR namaste[Title/Abstract] OR music[Title/Abstract] OR smell[Title/Abstract] OR aroma[Title/Abstract]) OR (“massage therapy”) OR (“sensory stimulation”) OR (“music therapy”) OR (“therapeutic touch”)) AND ((“dementia”) OR (“alzheimers”) OR (“end of life”) OR (“palliative”) OR (“coma”)) Filters: published in the last 10 years; Humans	
PubMed search 2 (run 26.4.17, terms relating to person-centred care)	
((“person centred care”) OR (“person centred care”[Title/Abstract]) OR (person centred care) OR ((“biography”) OR (biography[Title/Abstract] OR biographical[Title/Abstract]))) AND ((“residential care”) OR (“nursing home”) OR (“care home”) OR (“residential home”))	

#### Selection and appraisal of documents

Results of the searches were imported into bibliographic software. Two researchers independently screened the title and abstract of records (RS, JL, FB, CG) and the full text of articles that appeared to be relevant. Papers were assessed for inclusion on the basis of whether they were considered ‘good enough and relevant enough’ [24, 25]. This was an ongoing process that involved discussion between team members. Good enough was based on the reviewers’ assessment of whether the research was of a sufficient standard of its type and if the claims made were considered trustworthy. Papers were judged to be relevant if it was felt that the authors provided sufficient information and/or theoretical discussion to contribute to the programme theories being tested. Studies that were poorly conducted could still be included if the relevance was high, e.g. they contributed to our understanding about how a programme was thought to work.

#### Data extraction and synthesis

In Phase 1 we extracted information on how Namaste Care was interpreted and delivered, including the core components, and reported outcomes. In Phase 2 we extracted information on: study focus, participants, setting, intervention (including method of delivery and duration), how outcomes were measured and reported, and how underlying assumptions about the intervention were articulated. In a realist review data are not restricted to outcomes measured or results but also include author explanations. For example, discussions can provide a rich source of ‘data’ that helps explain how an intervention was thought to work (or why it did not). Data were extracted into an ACCESS database where the query feature was used to create tables enabling the identification of recurrent patterns of contexts and outcomes in the data and the possible mechanisms by which they occurred. [26] In addition, we mapped the most commonly reported outcomes (e.g. agitation) against data on context and mechanisms.

#### Testing and refining programme theory

To enhance the trustworthiness of our programme theory we held a second project team workshop (*n* = 7) to discuss the theory and undertook a second round of stakeholder consultation. This consultation involved discussion of the CMOs and was conducted via telephone interviews (*n* = 1), face-to-face (*n* = 2) and email (n = 1). In addition, findings from the review were presented to, and discussed with, a group of end of life care specialists (*n* = 40) at a community of practice meeting organized by specialist end of life and dementia care organisations. Many of those attending had direct experience of Namaste Care. Stakeholders were from similar groups as in Phase 1 (2 people took part in both sets of consultation).

## Results

### Description of included evidence

#### Phase 1

**Fig. 1:**
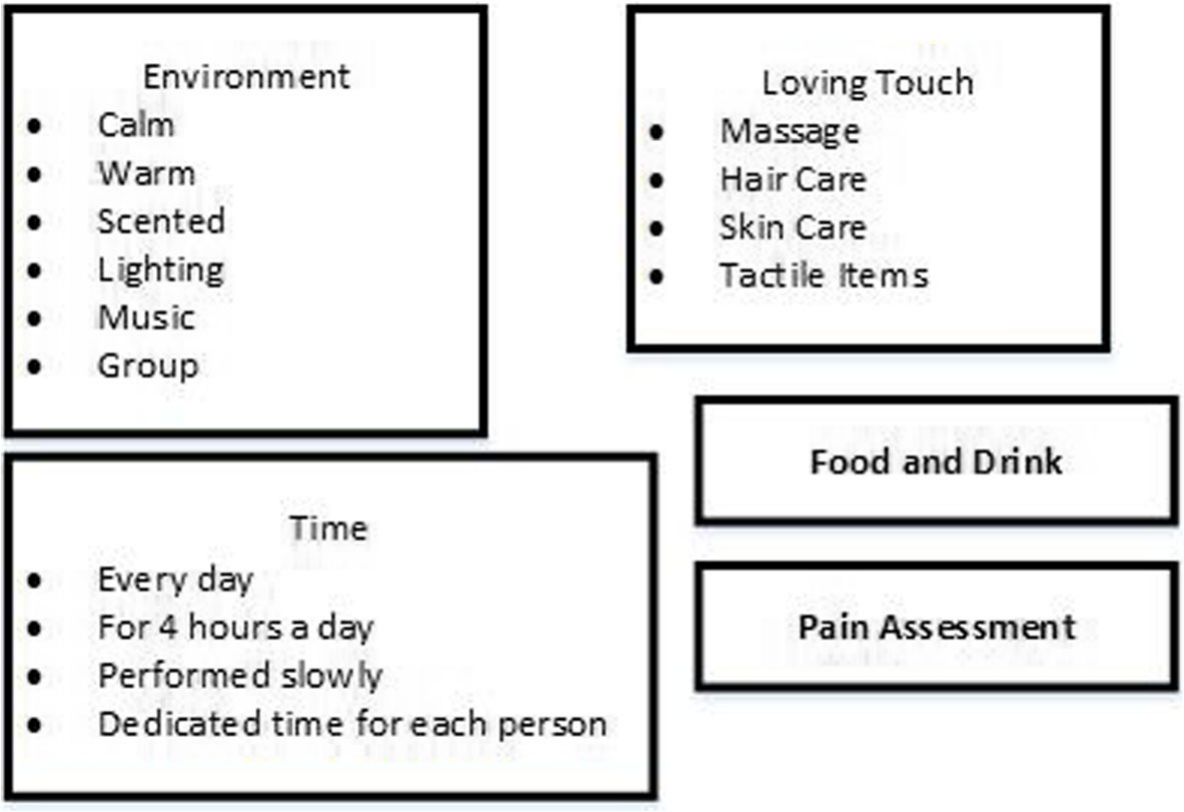
Elements of Namaste Care seen as core (from literature and from stakeholder accounts)

In Phase 1 we found 25 papers relating to Namaste Care, 18 of which provided sufficient information for theory development. The majority were descriptive accounts of Namaste Care rather than research studies. Of the seven research studies three included some before and after data [8, 27, 28], three were qualitative [29–31] and one (reported in three papers) used an action research approach [10, 12, 32]. Only five studies presented data on resident outcomes [8, 12, 13, 27, 28]. The seven research studies, and one further Namaste Care study identified during the Phase 2 searches [33], were taken forward for inclusion in Phase 2 (see Fig. 1). Core elements of Namaste Care, derived from the literature and stakeholder accounts are shown in Fig. 1 and details of the Namaste Care studies in Additional file 3.

#### Phase 2

**Fig. 2:**
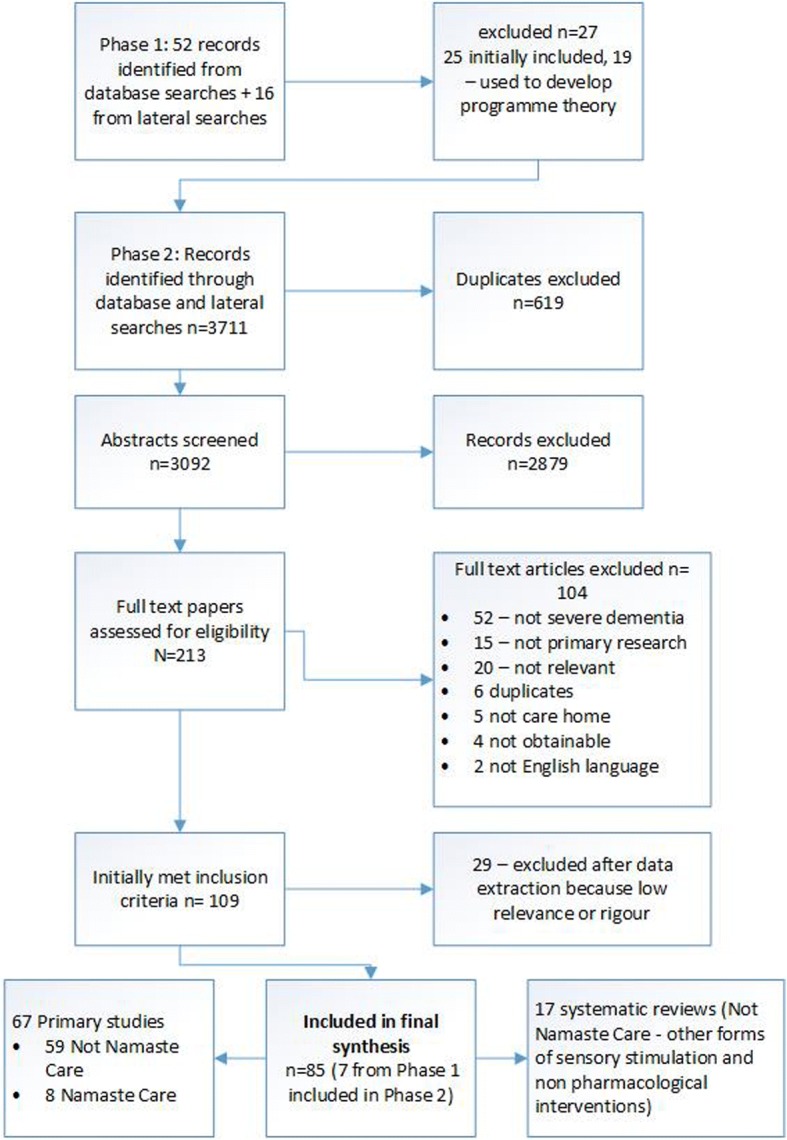
Overview of study selection process

In Phase 2 we included 85 papers. This comprised 17 evidence reviews (not Namaste Care) [34–50], 59 primary studies (not Namaste Care) [33, 51–109] and eight Namaste Care studies [8, 10, 12, 27–31]. The 59 primary papers included 24 Randomised controlled trials [59, 60, 64, 65, 70, 78, 79, 81–83, 87, 89, 90, 92–97, 99, 104, 106–108], five non randomized controlled studies [67, 74, 88, 100, 110], three before/after studies [43, 62, 95], ten observational studies [52–58, 66, 68, 109] and ten qualitative studies [71, 72, 76, 77, 80, 84, 85, 101–103]. The rest included a variety of study designs including pilot studies and cross over studies. Eight of the non-Namaste Care included studies [52–58, 109] were conducted by the same authors and relate to two datasets. The study selection process can be seen in Fig. 2, summary details of studies in Table 2, and further details of included studies in Additional file 4 and reviews in Additional file 5.

**Table 2:** Summary details of papers included in Phase 2

Number of studies			
	Category	Primary studies	Reviews
Main focus	Namaste Care	8	0
	Multisensory	21	0
	Music	18	6
	Touch/massage	10	1
	Aromatherapy	5	2
	Environment	7	2
	Other /mixed	10	6
Number of participants	• More than 200 participants n = 2 • More than 100 participants n = 12 (8 of which are the related Cohen-Mansfield studies) • Between 50 and 100 participants *n* = 12 • Under 50 participants *n* = 33		
Area where intervention was delivered	• Dedicated space e.g. Snoezelen room/multisensory environment/Namaste Care room *n* = 16 • Communal room (e.g. activities or dining room) *n* = 8 • Bedside/residents room *n* = 9 • Bathroom *n* = 2 • Not stated or not clear *n* = 24		
Intervention delivered by	• Researchers/researchers acting as facilitators *n* = 20 • Care or nursing home staff (e.g. care assistants or nurses) *n* = 21 • Music therapist n = 7 • Other therapists (e.g OT, physio) n = 5 • Snoezelen facilitator n = 2 • Sonas practitioner n = 2 • Activity coordinator n = 3 • Other *n* = 3 • Not clear n = 4		
Main outcomes measured	Resident outcomes • Agitation/behavioural symptoms *n* = 42 • Mood *n* = 13 • Alertness/improved communication n = 8 • Pain n = 3 Staff outcomes n = 12 (but most data anecdotal or qualitative)		
Country	• USA *n* = 17 • UK n = 13 • Australia n = 9 • Japan n = 5 • Taiwan n = 3 • Canada, Portugal, Norway, Sweden, Spain, Italy n = 2 in each • Netherlands, France, Belgium, Ireland n = 1 in each		

In the Namaste Care studies the programme was delivered by care home staff in five [27–30, 33], by Namaste Care carers in two [8, 12] and activity coordinators in one [31]. In other primary studies care home staff were involved in delivering the intervention in 14 studies [33, 63, 66, 68, 86, 88, 93, 97, 106, 107, 111]. In the rest it was unclear who delivered the intervention (*n* = 5) or the intervention was delivered by researchers or outside facilitators (e.g. music therapists). The most lengthy and frequent sessions were reported in the Namaste Care studies, with several reporting interventions delivered for four hours seven days a week [8, 12, 27]. In non Namaste Care studies the mean length of a session (provided in 12 studies) was 29 min. Fifteen studies specified that the intervention was group based, in 18 it was not clear, and, in the remainder, it was delivered one-to-one. The most commonly reported resident outcomes were related to behavioural symptoms, agitation or mood. Few staff outcomes were reported.

### Programme theory

**Fig. 3:**
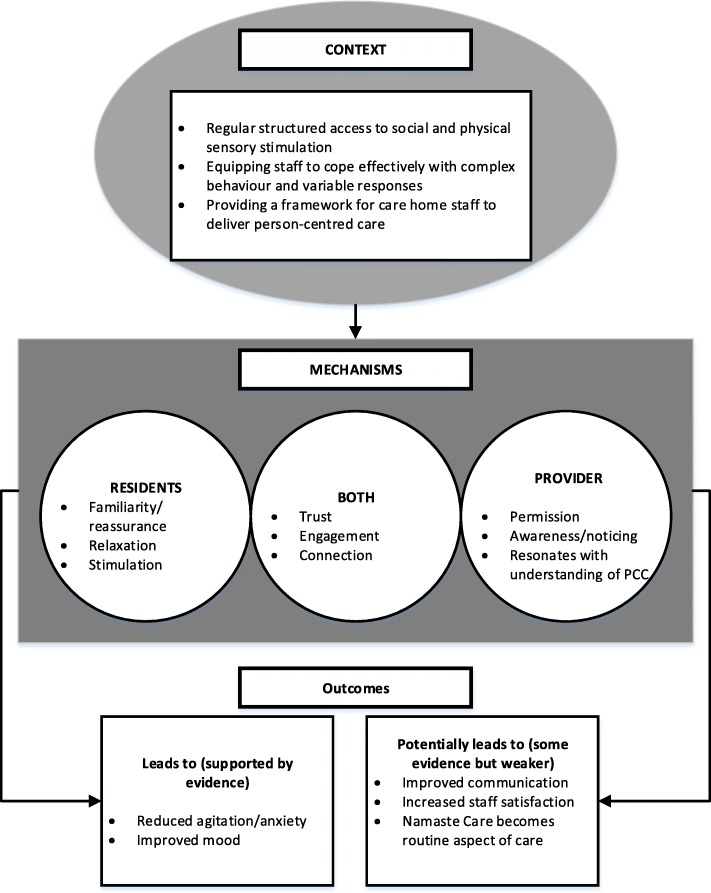
Summary of the three CMO configurations that make up the programme theory

Our review resulted in three context-mechanism-outcomes configurations which together provide an account of how and why Namaste Care might work for people with advanced dementia. These are presented in Table 3 and summarized in Fig. 3. Interventions were delivered by a variety of different occupational groups; we use the term provider to encompass all these groups. Quotes from stakeholders relating to the CMOs can be seen in Table 4.

**Table 3:** Overview of three final context-mechanism-outcome configuration

CMO1: Namaste Care provides regular, structured access to social and physical stimulation for people living with advanced dementia		
Context	Mechanism	Outcome
Namaste Care provides access to social and physical stimulation through a routine/structure consisting of: • Designated space • Considered timing • Regular skilled facilitator • Repetition of intervention • Social interaction • Regular drinks and snacks	• Residents: familiarity, consistency, reassurance, recognition • Trust develops between residents and carers • Namaste Care is seen by staff as a core activity • Staff are ‘intentionally present’ with residents • Staff given permission to engage	• Decrease in agitation/behavioural symptoms/anxiety • Improvements in mood • Intervention become part of care home routine • Staff satisfaction
CMO2: Namaste Care equips staff to work with people with complex behaviours and variable responses		
Context	Mechanism	Outcome
Namaste Care is delivered through a multi-sensory experience (effect) that provides a ‘toolkit’ of activities (resource) for staff	• Residents are soothed and relaxed by multi-sensory experience • Residents feel stimulated through multiple interactions • Staff notice/are more responsive to the needs of residents • Moments of connection are created between staff and residents	• Agitation/behavioural symptoms • Anxiety • Mood
CMO3: Namaste Care provides a framework for person-centred care		
Context	Mechanism	Outcome
Namaste Care focuses attention on residents’ biography and preferences (person-centred care)	• Resonates with staff’s understanding of person-centred care • Staff notice/are more aware of the needs of residents. • Trust develops between residents and carers	• Agitation • Mood • Engagement • Improved communication between staff and residents • Improved communication between residents and family & staff and family

**Table 4:** Supporting evidence from realist interviews with stakeholders

CMO1: Namaste Care provides regular, structured access to social and physical stimulation for people living with advanced dementia	
• ‘When you see proper results is when it’s a programme, when it happens 7 days a week … and that involves a huge change in the culture of the care home’ Nam03 • ‘But I think the skill in actually developing somebody’s understanding and application of Namaste [Care] is to take them down that journey, to help them connect and emotionally connect.’ Nam05 • ‘I think that’s a really big thing that Namaste [Care] adds is that there’s like structured time to really pay attention to the residents and yeah, and give them that extra time, extra, and also the opportunity to make contact with the residents.’ Nam09 • ‘[We] make them feel safe, make them feel comfortable, and then you see the results that we’re getting and I do think that’s the whole package, that’s the taste, that’s the smells, that’s the touch, that’s all of it.’ Nam01 • ‘I think that it’s very difficult for care home staff .. to be given space and time and energy and permission to do.’ Nam05 • ‘You just get to kind of focus and bond with the residents.’ Nam02 • ‘I think it …encourages them and gives them permission to find space for their residents… and the fact that everybody’s doing it makes it acceptable within the care home.’ Nam04 • ‘I think when Namaste [Care] really works is when you can create a space where people who are overstimulated will settle down, calm down and may go to sleep and that’s great but where people who are withdrawn can actually come out of that shell and can connect and make eye contact and maybe start to try and talk again’ Nam03 • ‘I think most of us are sort of social people, we don’t spend twenty four hours a day in a room … by bringing them out into a shared space they’re in a room that’s set up for that, they’ve got people around them, and I think the staff there do have good connections with their residents.’ Nam04 • ‘What they felt positive about was that they’d managed to create and access what they called a special atmosphere, an environment to practice Namaste Care.’ Nam06 • ‘what sort of ignited my passion was just seeing people sat and left for hours and hours in chairs and in beds and the only interaction that they would have would be to deliver a care task, so there is definitely a massive gap in care provision [that Namaste Care addresses].’ Nam10	
CMO2: Namaste Care equips staff to work with people with complex behaviours and variable responses	
• ‘I think watching staff I think what you see is that they realise that this person that may be End of Life, they may have really quite advanced dementia but we’re still reaching them… they’re still living Nam01 • ‘You go in there and you just see residents that won’t, you won’t ever engage with as such and they just kind of really come out of their shell and it is really satisfying after.’ Nam02 • ‘We’re a lot more involved with the residents and it’s a lot more, you can have a lot more conversation with them ..but obviously with the Namaste [Care] it’s kind of brought out of a lot of things in people we never saw.’ Nam02 • ‘People’s perception of going into a care home, where people were loud and screaming and shouting, and actually with Namaste Care in it was calm, people were relaxed, people were engaged with what they were doing.’ Nam04 • ‘Some days [the resident] liked it, and some days she didn’t, and so for me, the key is knowing the resident, which the staff did…but also this idea of, and I think more and more I’m becoming convinced of the importance of being mindfully attuned to how a person is responding on any given day.’ Nam06	
CMO3: Providing a framework for person-centred care	
• ‘a structure around these very human, very person-centred, very, uh, subjective things that you can do to engage with somebody that I would say good dementia care practitioners would do anyway, but it gives a sense of structure and permission for care staff to do this’ Nam 05 • ‘If I really had to say what I thought the main impact was, I think it’s the impact on the staff because we have our session and my residents go away and it’s gone but I have not lost the sense that actually, I can make you smile, I can do it and also that if I wait you may talk to me and once that gets into my head, why wouldn’t I use that when I’m trying to get you up and dressed or give you a bath or whatever else’ Nam03 • ‘I’ve got a lady at the moment, she’s completely, we have to do everything,… it’s just watching whether you get that spark of recognition in her eyes because that’s all you’re going to see, there is no other body language, but you’ll see her follow the lights with her eyes or she’ll open her mouth when you go to give her something, and that’s massive because somebody like that a few years ago would have just been left sat in a chair.’ Nam01 • ‘While you’re doing it and while you’re observing it you notice things that actually that might not work so well for that person … so it’s about thinking about your residents and how they’re changing and what we need to do to keep people involved.’ Nam01 • ‘it’s a lot of one-on-one time with the residents as well as in the groups.’ Nam02 • ‘you’ve got to have fundamentally good nursing care and the staff need to have good dementia care training as well,… but what I think Namaste [Care] does is to make it real for them, you know, it makes the person-centred care real for them and it then feeds into the basic care that they’re giving.’ Nam03 • ‘I think there’s something about setting up the Namaste Care programme, that people pay attention to people’s responses within the Namaste Care space.’ Nam04 • ‘The key to it was the capacity to offer interventions for the purpose of offering comfort, but facilitating opportunities for intentional presence and connection.’ Nam06 • ‘I hear people say that they feel that there’s this trickledown effect with Namaste [Care], …, that how they care for a person outside of the Namaste [Care] room is more caring.’ Nam07	

### CMO 1: Namaste care provides structured access to social and physical stimulation

- Programme theory: Care home interventions (e.g. Namaste Care) that provide regular and structured access to social and physical stimulation for residents with advanced dementia give staff permission to engage with residents outside of task-based care and improve resident outcomes (e.g. reduce agitation) through triggering responses such as familiarity, reassurance and trust.

The evidence suggests that one of the most important aspects of programmes is how they enable meaningful relationships to form between providers and residents, for example by having the same person provide each session, incorporating one to one interaction into an activity and providers having skills to work with people with advanced dementia [33, 40, 66, 70, 75, 79, 88, 101, 104, 112]. In contrast interventions involving providers who are unfamiliar to residents and/or do not have appropriate skills [82, 83] or who are unable to engage socially with people with dementia [111] may be less effective. Stakeholders at the workshop suggested that having the same person deliver Namaste Care was not always practical but rather the aim should be to achieve a consistent approach and attitude towards programme delivery.

Social stimulation appears to be a particularly key component of interventions. In a series of studies [52–58, 109] Cohen-Mansfield and colleagues evaluated a variety of stimuli for people with dementia living in care homes. They found that social stimulation, especially when it involved one to one interaction and the active participation of the resident, had the most dramatic effect on engagement and attention [56]. The importance of one to one attention and social stimulation was also highlighted by other studies [79, 87, 104, 106].

In Namaste Care physical stimulation is provided through both the components of the intervention and the environment in which the programme is delivered. For example, scents and soft music were felt to be calming and soothing for residents [8, 12, 29]. Stakeholders supported this suggesting that the right space could help over-stimulated residents relax and encourage people who were withdrawn to ‘emerge from their shell’. Namaste Care studies suggested that soft lighting was also an important part of the intervention [8, 28, 29, 31] but Cohen-Mansfield et al. found that normal levels of lighting (rather than bright or dark) were associated with pleasures in people with dementia [63]. Whilst some non Namaste Care studies referred to the importance of environment (for example having a private space or a quiet room) in many the space was not described (see Table 3). Studies however, did identify practical as well as therapeutic benefits to having a designated space, suggesting that sessions were less likely to be cancelled because of competing priorities and activities could take place as and when needed by the residents [75, 80]. However, whilst space might be an important context it is unlikely to trigger staff engagement without additional resources such as allocation of time and management support. A study evaluating the use of a sensory room for people with dementia (snoezelen) found that staff missed sessions because they did not see it as a priority [111]. Stakeholders suggested that for Namaste Care to be achievable and adopted as a core part of the work of the care home it was important that staff were given permission, through appropriate allocation of time and resources, to engage with it.

The originator of Namaste Care suggests that it should be delivered twice a day, seven days a week. Three non-randomised studies reported delivering Namaste Care in this way [8, 12, 27], although stakeholders suggested that this was unlikely to be feasible in most care homes in the UK. We found little empirical evidence on the optimal ‘dose’ of sensory interventions such as Namaste Care. The literature does, however, suggest that interventions that are delivered more regularly are important for creating a sense of reassurance and familiarity and building trusting relationships between residents and carers [35]. This in turn may help to reduce agitation and anxiety in people with advanced dementia. A meta-analysis of music therapy found that sessions done twice a week had a more statistically significant impact on disruptive behaviours, anxiety and mood than weekly sessions [34].

There was little evidence on the benefits of group versus one to one delivery. A meta-analysis of music therapy for people with dementia found group therapy had more positive effects on disruptive behaviours and anxiety than individual therapy [34]. However, this analysis did not distinguish between those with and without severe dementia. One RCT found group music therapy was more effective for residents with mild and moderate dementia than those with severe dementia [108] and another suggested that it was more difficult to achieve therapeutic goals if the ratio of participants to therapist was too high (e.g. five residents to one therapist) [104].

### CMO 2: Equipping staff to cope effectively with complex behaviours and variable responses

- Programme theory: Interventions that include a ‘toolkit’ of multisensory activities equip staff to work effectively with residents with complex behaviours and variable responses leading to improvements in resident outcomes (e.g. reduced agitation) through triggering responses such as engagement and connection between residents and carers.

The use of ‘loving touch’ is perceived to be key to Namaste Care, with touch thought to evoke an emotional response that leads to physical engagement [30]. We found some evidence to suggest that touch (such as hand massage) can have a calming effect [78] reduce behavioural symptoms [52, 100], improve sleep [69] and increase engagement [76]. Hand massage may be more effective than simulated social intervention (e.g. holding a doll) because the intervention combines one-to-one social interaction with sensory stimulation [109].

Music also appears to trigger emotional responses in people with advanced dementia. There is evidence that receptivity to music can remain until the late stages of dementia [48]. Primary studies [56, 68, 80, 89, 93] and reviews [34, 48] reported that music therapy improved communication and connection, increased engagement and reduced agitation [66, 68, 80, 112]. An advantage of music therapy is that it is inexpensive and easily implemented into care home activities [92].

There is some evidence to suggest that the most effective interventions are those that equip care staff to cope effectively with the complexity of caring for people with advanced dementia. A systematic review of interventions to reduce agitation in people with dementia found that the complexity of behaviour associated with dementia required a multifaceted response that could be tailored to the needs of individuals [40]. Stimulating a range of senses may be particularly important for people no longer able to verbalise [33], and as cognitive function deteriorates people with dementia can become very sensitive to sensory experiences [62, 63]. The multisensory nature of an intervention such as Namaste Care means that staff have a range of activities to draw on, giving residents choice in what is delivered and access to different stimulation (touch, auditory, olfactory and visual) [33, 40, 59, 62, 73] The assumption from this evidence is that it is the combined effect of being able to use a range of activities that triggers staff capacity and ability to respond to residents’ symptoms and behaviours and not individual activities.

### CMO 3: Providing a framework for person-centred care

- Programme theory: Multisensory interventions that focus attention on residents’ individual biographies and which attempt to connect with residents’ reality make staff more responsive to residents needs and lead to improvements in resident outcomes (such as increased responsiveness).

Many studies highlighted the importance of individually tailored interventions and acknowledging that different people would respond differently to the same stimuli [8, 36, 40, 50, 83]. Such personalisation included attention paid to the environment in which an intervention was delivered [110]; the music being played [66, 92, 99, 108]; the aroma [64, 65, 83, 88, 93, 102] the way someone was touched [82, 83, 88, 106] and how they were spoken to [76, 101]. It was also considered to be important to consider people’s known habits and preferences, the stage of dementia that people presented with and whether current preferences may be different to previous habits [76, 80].

Individualising interventions (e.g. using past and current preferences) was reported to reduce agitation [40, 52, 107] and increase alertness or engagement [57]. There was also evidence (although this was largely qualitative or anecdotal) that personalised interventions made staff notice more about residents and their abilities [10, 12, 33, 76], leading to improved communication between staff and residents [44, 50, 66, 70, 111],the development of trusting relationships between residents and caregivers [72, 75, 88] and a shift towards a more person-centred culture of care [10, 29]. In our original programme theory we hypothesised that Namaste Care would have benefits for family members, either through better connection with their family member with dementia or through improved communication with staff. Few studies measured outcomes for family members although there was some anecdotal and qualitative evidence that it improved connections between family members and residents [27, 31, 33, 67, 71, 76] and relatives and care home staff [27, 29, 33].

## Discussion

We have developed an explanation of how the Namaste Care intervention achieves particular outcomes, and in what circumstances. Our theory draws on three context-mechanism-outcome configurations that together provide an account of what needs to be in place for Namaste Care to work for people with advanced dementia. The CMOs highlight the importance of: delivering and structuring the intervention in a way that triggers feelings of familiarity and reassurance in residents, the involvement of providers with appropriate attitudes and skills, one-to-one engagement, and the incorporation of resident biographies and preferences into programme delivery. It also highlights the need to provide appropriate preparation and resources for staff to engage with the programme. A key overarching mechanism that emerged concerned the importance of activities that enabled the development of moments of connection for people with advanced dementia. This might be connection with a provider or member of staff, with a family member or with a memory through an activity such as music.

### Implication of findings

The creators of Namaste Care proposed that it is delivered regularly and intensively, ideally twice a day, seven days a week [9]. However, the evidence to support this is limited. We found only three non randomised studies evaluating an intervention of a similar intensity [8, 12, 27], and, whilst studies compared different active interventions none compared different doses of the same intervention. Moreover, stakeholders involved in delivering Namaste Care in care home settings suggested that delivering the intervention as originally intended is unlikely to be possible in many care homes. What our review does suggest is regular access to social and physical stimulation for people with advanced dementia triggers mechanisms such as familiarity, reassurance and recognition, which in turn leads to a reduction in agitation and behavioural symptoms. Regular engagement in multisensory interventions may be important as the evidence from the review, and from the wider literature, suggests that the impact on outcomes such as agitation may be short-lived [36, 52, 113].

Namaste Care is designed to be delivered to a group, albeit involving individual attention for residents. This is in part to reduce the isolation of care home residents with dementia by bringing them together to experience Namaste Care in a communal room. It was also considered preferable in terms of staffing levels [9]. From our review there was insufficient evidence to say whether group delivery had therapeutic benefits or if it is just a more practical way of delivering an intervention in a care home. What does seem to be an essential context is the opportunity for meaningful one to one interaction between residents and carers [114]. This interaction was more likely to create moments of connection if the provider had appropriate skills and was comfortable in social interactions with residents with advanced dementia. This review included studies looking at a variety of sensory interventions, including those that involved multiple activities and those that were focused on a single activity. The work by Cohen-Mansfield and colleagues [56–58] suggests that, whilst any type of stimulus is preferable to none, there may be a hierarchy of sensory stimuli with live social stimuli (involving one to one interaction) being most effective.

Our theory suggests that one of the ways in which Namaste Care works is by providing a clear structure that gives staff both permission, and opportunity, to deliver person centred care. Previous research has found a person centred approach to dementia care is associated with improved outcomes for care home residents [107, 113, 115, 116]. In this review we found evidence to suggest that using biography and personal preferences were important contextual features of multisensory interventions because they helped to trigger the creation of connections for residents. As Namaste Care includes a range of activities we suggest that it is easier for staff to find activities that fit with individual preferences and responses. In our initial programme theory we hypothesised that being involved in the delivery of Namaste Care would improve staff outcomes, for example increasing satisfaction and confidence. Few studies measured these outcomes but it remains a plausible idea. Studies have found a positive association between implementing person-centred care strategies and a sense of staff confidence [117] and satisfaction [118].

In developing our programme theory we focused primarily on Namaste Care rather than the wider care home environment. However, there is good evidence that organisational context in long-term care settings affects the uptake of healthcare innovation [119]. Important contextual elements are alignment with care home priorities, senior management support at organisational and unit level, and care home staff having enough slack and flexibility to accommodate change into current workload. Namaste Care may require additional resources, particularly staff input, in the start-up phase [120]. All of these are important in order for staff to feel that Namaste Care is a priority and part of the everyday work of the care home [12].

### Strengths and limitations

There is a lack of research evaluating the impact of Namaste Care. However, the advantage of a realist approach is that the unit of analysis is the programme theory, or underpinning mechanism of action, rather than the intervention [121]. This meant we were able to include a broader range of literature, such as interventions drawing on similar principles to Namaste Care. This provided opportunities for transferable learning and enabled us to develop a theory driven explanation that can be used to guide future initiatives and evaluations. There remain, however, unanswered questions about the effectiveness of Namaste Care. For example, we do not know what the impact is of involving family members in Namaste Care (or if it is feasible), whether food and drink are important, whether there is a therapeutic effect of group delivery, whether some stimuli are more effective than others, or how often Namaste Care needs to be delivered to be effective. Some of these questions will be explored further in a feasibility trial currently underway [122].

In realist reviews the aim is not so much to summarise all the available evidence but rather to make sense of it. Searching tends, therefore, to be iterative and ongoing throughout the review process with the aim of identifying sufficient sources for theory building and testing. To identify studies for our review we undertook extensive database and lateral searching. Despite this it is possible that we missed potentially relevant literature. However, the nature of realist methodology means that there is not a finite set of relevant papers to be found. Instead the reviewer can take a more purposive approach to sampling that aims to reach conceptual saturation rather than identify all available documents [121].

This review is the first Phase of a study assessing the feasibility of Namaste Care as an intervention for improving the quality of end of life care in care homes. Whilst some of the Namaste Care studies suggest that Namaste Care can support good pain management in people with advanced dementia [10, 12] few of the studies we included focused on end of life care or reported pain as an outcome. This is in line with other research which has found that there are few interventions which address the challenges that dying with dementia poses [123]. Therefore, although we were able to develop a theory about how Namaste Care may improve quality of life for people with advanced dementia we do not know if, or how, it might improve end of life care. Our feasibility study will explore this further [122].

## Conclusions

Our realist review has shown that it is possible, using the available evidence, to develop and test a coherent account of how Namaste Care and similar multi-sensory interventions might work for people with advanced dementia living in long-term care settings. This review is important because it provides both practitioners and researchers with a framework to judge the feasibility and likely success of Namaste Care in long term settings. The proposed theoretical account of what works, why and in what circumstances is not final. As further relevant evidence emerges, it will be refined, challenged and developed further. Nevertheless, it is reasonable to conclude that the key mechanisms that Namaste Care triggers for residents are feelings of familiarity, reassurance, engagement and connection, and that for staff it gives them permission and awareness to engage with residents in a more person-centred way.

## Additional files

Additional file 1: Topic guide for stakeholder interviews. (PDF 84 kb)

Additional file 2: Development of Preliminary Programme Theory. (PDF 227 kb)

Additional file 3: Table of included Namaste Care studies. (PDF 271 kb)

Additional file 4: Table of included primary studies Phase 2. (PDF 277 kb)

Additional file 5: Table of included reviews Phase 2. (PDF 205 kb)

## Abbreviations

CMO: Context-mechanism-outcomes
RAMESES: Realist and meta-narrative evidence syntheses: evolving standards

## References

[1] Rait G, Walters K, Bottomley C, Petersen I, Iliffe S, Nazareth I (2010). Survival of people with clinical diagnosis of dementia in primary care: cohort study. BMJ.

[2] Alzheimer’s Society. Fix dementia care NHS and care homes. 2016.

[3] Department of Health. Dementia: a state of the nation report on dementia care and support in England. 2013; November:68. https://assets.publishing.service.gov.uk/government/uploads/system/uploads/attachment_data/file/262139/Dementia.pdf.

[4] Sclan SG, Reisberg B (1992). Functional assessment staging (FAST) in Alzheimer’s disease: reliability, validity, and Ordinality. Int Psychogeriatrics.

[5] Reisberg B, Ferris SH, de Leon MJ, Crook T (1982). The global deterioration scale for assessment of primary degenerative dementia. Am J Psychiatry.

[6] Murphy E, Froggatt K, Connolly S, Shea OE, El S, Casey D, et al. Palliative care interventions in advanced dementia. Cochrane Database Syst Rev. 2016;(12):1–48. Art. No.: CD011513. 10.1002/14651858.CD011513.pub2 PMC6463843 27911489

[7] Amador S, Goodman C, King D, Ng YT, Elmore N, Mathie E (2014). Exploring resource use and associated costs in end-of-life care for older people with dementia in residential care homes. Int J Geriatr Psychiatry..

[8] Simard J, Volicer L (2010). Effects of namaste care on residents who do not benefit from usual activities. Am J Alzheimer’s Dis Other Dementias.

[9] Simard J (2013). The end-of-life Namaste care program for people with dementia. Second Edi.

[10] Stacpoole M, Hockley J, Thompsell A, Simard J, Volicer L (2017). Implementing the Namaste care program for residents with advanced dementia: exploring the perceptions of families and staff in UK care homes. Ann Palliat Med.

[11] Stacpoole M, Thompsell A (2015). OA25 The namaste care programme can enrich quality of life for people with advanced dementia and those who care for them without additional resources. BMJ Support Palliat Care.

[12] Stacpoole M, Hockley J, Thompsell A, Simard J, Volicer L (2015). The Namaste care programme can reduce behavioural symptoms in care home residents with advanced dementia. Int J Geriatr Psychiatry.

[13] Fullarton J, Volicer L (2013). Reductions of antipsychotic and hypnotic medications in Namaste care. J Am Med Dir Assoc.

[14] Rycroft-Malone J, McCormackB, Hutchinson AM, DeCorby K, Bucknall TK, Kent B, Schultz A, Snelgrove-Clarke E et al. Realist synthesis: illustrating the method for implementation. 2012;7:33. 10.1186/1748-5908-7-33research. 10.1186/1748-5908-7-33 PMC3514310 22515663

[15] Wong G, Greenhalgh T, Westhorp G, Buckingham J, Pawson R (2013). RAMESES publication standards: realist syntheses. BMC Med.

[16] Pawson R, Greenhalgh T, Harvey G, Walshe KR (2004). Realist synthesis: an introduction.

[17] Pawson R, Greenhalgh T, Harvey G, Walshe K (2005). Realist review a new method of systematic review designed for complex policy interventions. J Heal Serv Res Policy.

[18] Pawson R, Walshe K, Greenhalgh T (2004). Realist synthesis : an introduction.

[19] Pawson R (2006). Evidence-based policy: a realist perspective.

[20] Manzano A (2016). The craft of interviewing in realist evaluation. Evaluation.

[21] Pearson M, Brand SL, Quinn C, Shaw J, Maguire M, Michie S (2015). Using realist review to inform intervention development: methodological illustration and conceptual platform for collaborative care in offender mental health. Implement Sci.

[22] Bartunek JM, Murninghan JK (1984). The nominal group technique: expanding the basic procedure and underlying assumptions. Gr Organ Manag.

[23] Ford JA, Wong G, Jones AP, Steel N. Access to primary care for socioeconomically disadvantaged older people in rural areas: a realist review. BMJ Open. 2016;6:e010652. 10.1136/bmjopen-2015-010652 PMC4874140 27188809

[24] Rycroft-Malone J, Burton C, Hall B, McCormack B, Nutley S, Seddon D (2014). Improving skills and care standards in the support workforce for older people: a realist review. BMJ Open.

[25] Pawson R (2002). Evidence-based policy: the promise of ‘realist synthesis. Evaluation.

[26] Wong G, Pawson R, Owen L (2011). Policy guidance on threats to legislative interventions in public health: a realist synthesis. BMC Public Health.

[27] Manzar B, Volicer L (2015). Effects of Namaste care: pilot study. Am J Alzheimers Dis.

[28] Soliman A, Hirst S (2015). Using sensory activities to improve dementia care. Nurs Times.

[29] McNiel P, Westphal J. Namaste care : a person-centered care approach for Alzheimers and advanced dementia. West J Nurs Res. 2016; 40(1):37-51. 10.1177/0193945916679631 27885156

[30] Nicholls D, Chang E, Johnson A, Edenborough M (2013). Touch, the essence of caring for people with end-stage dementia: a mental health perspective in Namaste care. Aging Ment Health.

[31] John KS, Koffman J. Acceptability of Namaste Care for patients with advanced dementia being cared for in an acute hospital setting. 2015;:1–13.

[32] Stacpoole M, Thompsell A, Hockley J, Christophers S. Toolkit for implementing the Namaste Care programme for people with advanced dementia living in care homes. 2016. https://www.stchristophers.org.uk/wp-content/uploads/2016/03/Namaste-Care-Programme-Toolkit-06.04.2016.pdf. Accessed 24 Jan 2017.

[33] Magee M, Mccorkell G, Guille S, Coates V (2017). Feasibility of the Namaste care Programme to enhance care for those with advanced dementia. Int J Palliat Care Nurs.

[34] Chang YS, Chu H, Yang CY, Tsai JC, Chung MH, Liao YM (2015). The efficacy of music therapy for people with dementia: a meta-analysis of randomised controlled trials. J Clin Nurs.

[35] Chatterton W, Baker F, Morgan K (2010). The singer or the singing: who sings individually to persons with dementia and what are the effects?. Am J Alzheimers Dis Other Demen.

[36] Livingston G, Kelly L, Lewis-Holmes E, Baio G, Morris S, Patel N (2014). A systematic review of the clinical effectiveness and cost-effectiveness of sensory, psychological and behavioural interventions for managing agitation in older adults with dementia. Heal Technol Assess.

[37] Nguyen QA, Paton C (2008). The use of aromatherapy to treat behavioural problems in dementia. Int J Geriatr Psychiatry.

[38] Padilla R, Domina A (2016). Effectiveness of sensory stimulation to improve arousal and alertness of people in a coma or persistent vegetative state after traumatic brain injury: a systematic review. Am J Occup Ther.

[39] Raglio A, Filippi S, Bellandi D, Stramba-Badiale M (2014). Global music approach to persons with dementia: evidence and practice. Clin Interv Aging.

[40] Randall EW, Clissett PC (2015). What are the relative merits of interventions used to reduce the occurrences of disruptive vocalisation in persons with dementia? - a systematic review. Int J Older People Nursing.

[41] Wall M, Duffy A (2010). The effects of music therapy for older people with dementia. Br J Nurs.

[42] Wu J, Wang Y, Wang Z. The effectiveness of massage and touch on behavioral and psychological symptoms of dementia: a quantitative systematic review and meta-analysis. J Adv Nurs. 2017;73:2283–95. 10.1111/jan.13311 28378347

[43] Clare L (2010). Awareness in people with severe dementia: review and integration. Aging Ment Heal..

[44] Conn DK, Seitz DP (2010). Advances in the treatment of psychiatric disorders in long-term care homes. Curr Opin Psychiatry.

[45] Enmarker I, Olsen R, Hellzen O (2011). Management of person with dementia with aggressive and violent behaviour: a systematic literature review. Int J Older People Nursing.

[46] Ennis EM, Kazer MW (2013). The role of spiritual nursing interventions on improved outcomes in older adults with dementia. Holist Nurs Pr.

[47] Forrester LT, Maayan N, Orrell M, Spector AE, Buchan LD, Soares-Weiser K. Aromatherapy for dementia. Cochrane Database Syst Rev. 2014;(2):Cd003150. 10.1002/14651858.CD003150.pub2. 10.1002/14651858.CD003150.pub2 24569873

[48] Guetin S, Charras K, Berard A, Arbus C, Berthelon P, Blanc F (2013). An overview of the use of music therapy in the context of Alzheimer’s disease: a report of a French expert group. Dement.

[49] Kim SY, Yoo EY, Jung MY, Park SH, Park JH (2012). A systematic review of the effects of occupational therapy for persons with dementia: a meta-analysis of randomized controlled trials. NeuroRehabilitation.

[50] McDermott O, Crellin N, Ridder HM, Orrell M (2013). Music therapy in dementia: a narrative synthesis systematic review. Int J Geriatr Psychiatry.

[51] Anderson JG, Taylor AG (2012). Use of complementary therapies for cancer symptom management: results of the 2007 National Health Interview Survey. J Altern Complement Med.

[52] Cohen-Mansfield J, Marx MS, Dakheel-Ali M, Regier NG, Thein K, Freedman L (2010). Can agitated behavior of nursing home residents with dementia be prevented with the use of standardized stimuli?. J Am Geriatr Soc.

[53] Cohen-Mansfield J, Marx MS, Dakheel-Ali M, Thein K (2015). The use and utility of specific nonpharmacological interventions for behavioral symptoms in dementia: an exploratory study. Am J Geriatr Psychiatry.

[54] Cohen-Mansfield J, Marx MS, Freedman LS, Murad H, Regier NG, Thein K (2011). The comprehensive process model of engagement. Am J Geriatr Psychiatry.

[55] Cohen-Mansfield J, Marx MS, Freedman LS, Murad H, Thein K, Dakheel-Ali M (2012). What affects pleasure in persons with advanced stage dementia?. J Psychiatr Res.

[56] Cohen-Mansfield J, Marx MS, Thein K, Dakheel-Ali M (2011). The impact of stimuli on affect in persons with dementia. J Clin Psychiatry.

[57] Cohen-Mansfield J, Marx MS, Thein K, Dakheel-Ali M (2010). The impact of past and present preferences on stimulus engagement in nursing home residents with dementia. Aging Ment Heal.

[58] Cohen-Mansfield J, Thein K, Marx MS, Dakheel-Ali M, Murad H, Freedman LS (2012). The relationships of environment and personal characteristics to agitated behaviors in nursing home residents with dementia. J Clin Psychiatry.

[59] Collier L (2008). The use of multi-sensory stimulation to improve functional performance in older people with dementia: a randomised single blind trial. Br J Occup Ther.

[60] Collier L, McPherson K, Ellis-Hill C, Staal J, Bucks R (2010). Multisensory stimulation to improve functional performance in moderate to severe dementia—interim results. Am J Alzheimer’s Dis Other Dementias®.

[61] Cruz J, Marques A, Barbosa A, Figueiredo D, Sousa LX (2013). Making sense(s) in dementia: a multisensory and motor-based group activity program. Am J Alzheimers Dis Other Demen.

[62] Behrman S, Chouliaras L, Ebmeier KP (2014). Considering the senses in the diagnosis and management of dementia. Maturitas.

[63] Cruz J, Marques A, Barbosa AL, Figueiredo D, Sousa L (2011). Effects of a motor and multisensory-based approach on residents with moderate-to-severe dementia. Am J Alzheimer’s Dis Other Dementias.

[64] Fu CY, Moyle W, Cooke M (2013). A randomised controlled trial of the use of aromatherapy and hand massage to reduce disruptive behaviour in people with dementia. BMC Complement Altern Med.

[65] Fujii M, Hatakeyama R, Fukuoka Y, Yamamoto T, Sasaki R, Moriya M (2008). Lavender aroma therapy for behavioral and psychological symptoms in dementia patients. Geriatr Gerontol Int.

[66] Gotell E, Brown S, Ekman SL (2009). The influence of caregiver singing and background music on vocally expressed emotions and moods in dementia care: a qualitative analysis. Int J Nurs Stud.

[67] Goto S, Kamal N, Puzio H, Kobylarz F, Herrup K (2014). Differential responses of individuals with late-stage dementia to two novel environments: a multimedia room and an interior garden. J Alzheimers Dis.

[68] Hammar LM, Emami A, Gotell E, Engstrom G (2011). The impact of caregivers’ singing on expressions of emotion and resistance during morning care situations in persons with dementia: an intervention in dementia care. J Clin Nurs.

[69] Harris M, Richards KC, Grando VT (2012). The effects of slow-stroke back massage on minutes of nighttime sleep in persons with dementia and sleep disturbances in the nursing home: a pilot study. J Holist Nurs.

[70] Hsu MH, Flowerdew R, Parker M, Fachner J, Odell-Miller H. Individual music therapy for managing neuropsychiatric symptoms for people with dementia and their carers: a cluster randomised controlled feasibility study. BMC Geriatr. 2015;15:84. 10.1186/s12877-015-0082-4 PMC4506459 26183582

[71] Kellett U, Moyle W, McAllister M, King C, Gallagher F (2010). Life stories and biography: a means of connecting family and staff to people with dementia. J Clin Nurs.

[72] Kupeli N, Leavey G, Moore K, Harrington J, Lord K, King M (2016). Context, mechanisms and outcomes in end of life care for people with advanced dementia. BMC Palliat Care.

[73] Belgrave M (2009). The effect of expressive and instrumental touch on the behavior states of older adults with late-stage dementia of the Alzheimer’s type and on music therapist’s perceived rapport. J Music Ther.

[74] Lancioni GE, O’Reilly MF, Singh NN, Sigafoos J, Grumo G, Pinto K (2013). Assessing the impact and social perception of self-regulated music stimulation with patients with Alzheimer’s disease. Res Dev Disabil.

[75] Lape JE (2009). Using a multisensory environment: to decrease negative behaviors in clients with dementia. OT Pract.

[76] Litchke LG, Hodges JS (2014). The meaning of “now” moments of engagement in yoga for persons with Alzheimer’s disease. Ther Recreat J.

[77] Lykkeslet E, Gjengedal E, Skrondal T, Storjord MB (2014). Sensory stimulation - a way of creating mutual relations in dementia care. Int J Qual Stud Heal Well-being.

[78] Mariko A, Matsuda H, Takahashi M, Fujii M, Sasaki H (2015). Touch on the acupoint of Shinchuu of Alzheimer’s disease patients. Geriatr Gerontol Int.

[79] Maseda A, Sánchez A, Marante MP, González-Abraldes I, Buján A, Millán-Calenti JC (2014). Effects of multisensory stimulation on a sample of institutionalized elderly people with dementia diagnosis: a controlled longitudinal trial. Am J Alzheimers Dis Other Demen.

[80] McDermott O, Orrell M, Ridder HM (2014). The importance of music for people with dementia: the perspectives of people with dementia, family carers, staff and music therapists. Aging Ment Heal..

[81] Milev RV, Kellar T, McLean M, Mileva V, Luthra V, Thompson S (2008). Multisensory stimulation for elderly with dementia: a 24-week single-blind randomized controlled pilot study. Am J Alzheimers Dis Other Demen.

[82] Moyle W, Cooke ML, Beattie E, Shum DH, O’Dwyer ST, Barrett S (2014). Foot massage and physiological stress in people with dementia: a randomized controlled trial. J Altern Complement Med.

[83] Moyle W, Cooke ML, Beattie E, Shum DHK, O’Dwyer ST, Barrett S (2014). Foot massage versus quiet presence on agitation and mood in people with dementia: a randomised controlled trial. Int J Nurs Stud.

[84] Bergland A, Johansen H, Sellevold GS (2015). A qualitative study of professional caregivers’ perceptions of processes contributing to mealtime agitation in persons with dementia in nursing home wards and strategies to attain calmness. Nurs Open.

[85] Murphy JL, Holmes J, Brooks C (2017). Nutrition and dementia care: developing an evidence-based model for nutritional care in nursing homes. BMC Geriatr.

[86] Nair BK, Heim C, Krishnan C, D’Este C, Marley J, Attia J (2011). The effect of baroque music on behavioural disturbances in patients with dementia. Australas J Ageing.

[87] Narme P, Clement S, Ehrle N, Schiaratura L, Vachez S, Courtaigne B (2014). Efficacy of musical interventions in dementia: evidence from a randomized controlled trial. J Alzheimers Dis.

[88] Quell R, Skovdal K, Kihlgren M, Lökk J (2008). Using tactile stimulation in a dementia care facility with plasma prolactin as an outcome measure -- a pilot study. Arch Int J Med.

[89] Raglio A, Bellandi D, Baiardi P, Gianotti M, Ubezio MC, Zanacchi E (2015). Effect of active music therapy and individualized listening to music on dementia: a multicenter randomized controlled trial. J Am Geriatr Soc.

[90] Raglio A, Bellelli G, Traficante D, Gianotti M, Ubezio MC, Gentile S (2012). Addendum to “efficacy of music therapy treatment based on cycles of sessions: a randomised controlled trial” (Raglio et al., 2010). Aging Ment Heal.

[91] Rodriguez-Mansilla J, Gonzalez Lopez-Arza MV, Varela-Donoso E, Montanero-Fernandez J, Gonzalez Sanchez B, Garrido-Ardila EM (2015). The effects of ear acupressure, massage therapy and no therapy on symptoms of dementia: a randomized controlled trial. Clin Rehabil.

[92] Sakamoto M, Ando H, Tsutou A (2013). Comparing the effects of different individualized music interventions for elderly individuals with severe dementia. Int Psychogeriatr.

[93] Sakamoto Y, Ebihara S, Ebihara T, Tomita N, Toba K, Freeman S (2012). Fall prevention using olfactory stimulation with lavender odor in elderly nursing home residents: a randomized controlled trial. J Am Geriatr Soc.

[94] Sanchez A, Maseda A, Marante-Moar MP, de Labra C, Lorenzo-Lopez L, Millan-Calenti JC (2016). Comparing the effects of multisensory stimulation and individualized music sessions on elderly people with severe dementia: a randomized controlled trial. J Alzheimers Dis.

[95] Burns A, Perry E, Holmes C, Francis P, Morris J, Howes M-JR (2011). A double-blind placebo-controlled randomized trial of Melissa officinalis oil and donepezil for the treatment of agitation in Alzheimer’s disease. Dement Geriatr Cogn Disord.

[96] Staal JA, Sacks A, Matheis R, Collier L, Calia T, Hanif H, et al. The effects of Snoezelen (multi-sensory behavior therapy) and psychiatric care on agitation, apathy, and activities of daily living in dementia patients on a short term geriatric psychiatric inpatient unit. Int J Psychiatry Med. 2007;37:357–70. 10.2190/PM.37.4.a 18441625

[97] Strøm BS, Engedal K, Benth JS, Grov EK (2017). Effect of the sonas programme on communication in people with dementia: a randomized controlled trial. Dement Geriatr Cogn Dis Extra.

[98] Sung HC, Chang AM, Lee WL (2010). A preferred music listening intervention to reduce anxiety in older adults with dementia in nursing homes. J Clin Nurs.

[99] Sung HC, Lee WL, Li TL, Watson R (2012). A group music intervention using percussion instruments with familiar music to reduce anxiety and agitation of institutionalized older adults with dementia. Int J Geriatr Psychiatry..

[100] Suzuki M, Tatsumi A, Otsuka T, Kikuchi K, Mizuta A, Makino K (2010). Physical and psychological effects of 6-week tactile massage on elderly patients with severe dementia. Am J Alzheimers Dis Other Demen.

[101] Tuckett AG, Hodgkinson B, Rouillon L, Balil-Lozoya T, Parker D (2015). What carers and family said about music therapy on behaviours of older people with dementia in residential aged care. Int J Older People Nursing.

[102] Van Vracem M, Spruytte N, Declercq A, Van Audenhove C (2016). Agitation in dementia and the role of spatial and sensory interventions: experiences of professional and family caregivers. Scand J Caring Sci.

[103] Vezina A, Robichaud L, Voyer P, Pelletier D (2011). Identity cues and dementia in nursing home intervention. Work.

[104] Vink AC, Zuidersma M, Boersma F, de Jonge P, Zuidema SU, Slaets JP (2013). The effect of music therapy compared with general recreational activities in reducing agitation in people with dementia: a randomised controlled trial. Int J Geriatr Psychiatry.

[105] Ward-Smith P, Llanque SM, Curran D (2009). The effect of multisensory stimulation on persons residing in an extended care facility. Am J Alzheimers Dis Other Demen.

[106] Cameron H, du Toit S, Richard G, Bearns L (2011). Using lemon balm oil to reduce aggression and agitation in dementia: results of a pilot study. J Dement Care.

[107] Chenoweth L, King MT, Jeon Y-H, Brodaty H, Stein-Parbury J, Norman R (2009). Caring for aged dementia care resident study (CADRES) of person-centred care, dementia-care mapping, and usual care in dementia: a cluster-randomised trial. Lancet Neurol.

[108] Chu H, Yang CY, Lin Y, Ou KL, Lee TY, O’Brien AP (2014). The impact of group music therapy on depression and cognition in elderly persons with dementia: a randomized controlled study. Biol Res Nurs.

[109] Cohen-Mansfield J, Dakheel-Ali M, Marx MS, Thein K, Regier NG (2015). Which unmet needs contribute to behavior problems in persons with advanced dementia?. Psychiatry Res.

[110] Bicket MC, Samus QM, McNabney M, Onyike CU, Mayer LS, Brandt J (2010). The physical environment influences neuropsychiatric symptoms and other outcomes in assisted living residents. Int J Geriatr Psychiatry.

[111] Anderson K, Bird M, Macpherson S, McDonough V, Davis T (2011). Findings from a pilot investigation of the effectiveness of a snoezelen room in residential care: should we be engaging with our residents more?. Geriatr Nurs.

[112] Raglio A, Bellelli G, Traficante D, Gianotti M, Ubezio MC, Gentile S (2010). Efficacy of music therapy treatment based on cycles of sessions: a randomised controlled trial. Aging Ment Heal.

[113] Kim SK, Park M (2017). Effectiveness of person-centered care on people with dementia: a systematic review and meta-analysis. Clin Interv Aging.

[114] Travers C, Brooks D, Hines S, O’Reilly M, McMaster M, He W (2016). Effectiveness of meaningful occupation interventions for people living with dementia in residential aged care: a systematic review. JBI Database Syst Rev Implement reports.

[115] Brownie S, Nancarrow S (2013). Effects of person-centered care on residents and staff in aged-care facilities: a systematic review. Clin Interv Aging.

[116] Sloane PD, Hoeffer B, Mitchell CM, McKenzie DA, Barrick AL, Rader J (2004). Effect of person-centered showering and the towel bath on bathing-associated aggression, agitation, and discomfort in nursing home residents with dementia: a randomized, controlled trial. J Am Geriatr Soc.

[117] Mullan MA, Sullivan KA (2016). Positive attitudes and person-centred care predict of sense of competence in dementia care staff. Aging Ment Heal.

[118] Berendonk C, Kaspar R, Bär M, Hoben M. Improving quality of work life for care providers by fostering the emotional well-being of persons with dementia: A cluster-randomized trial of a nursing intervention in German long-term care settings. Dementia. 2017;0(0):1-24. 10.1177/1471301217698837. 10.1177/1471301217698837 29149793

[119] Goodman C, Sharpe R, Russell C, Meyer J, Gordon A, Dening T, Corazzini T, Lynch J BF. Care home readiness: a rapid review and consensus workshops on how organisational context affects care home engagement with health care innovation. 2017.

[120] Smaling H, Joling K, Achterberg W, Franke AL van der SJ. The Namaste Care Family program for people with advanced dementia: first experiences of staff and family caregivers. In: 24NKG. 2018.

[121] Pawson R, Greenhalgh T, Harvey G, Walshe K (2005). Realist review--a new method of systematic review designed for complex policy interventions. J Health Serv Res Policy.

[122] Froggatt K, Walshe C, Burnside G, Perez Algorta G, Kinley J, Preston N HB et al. The Namaste care intervention to improve the quality of dying for people with advanced dementia living in care homes: a realist review and feasibility study for a cluster randomised controlled trial. BMJ Open. 2018;8:e026531. 10.1136/bmjopen-2018-026531 PMC6254402 30478131

[123] Goodman C, Evans C, Wilcock J, Froggatt K, Drennan V, Sampson E (2010). End of life care for community dwelling older people with dementia: an integrated review. Int J Geriatr Psychiatry..

