# Supplementary material for: Improving living and dying for people with advanced dementia living in care homes: a realist review of Namaste Care and other multisensory interventions
Source: BMC Geriatr. 2018 Dec 6;18:303. doi: 10.1186/s12877-018-0995-9 (PMC6282262; doi:10.1186/s12877-018-0995-9)
Supplement: Supplementary file 1 — Topic guide for stakeholder interviews. (PDF 84 kb) [file 12877_2018_995_MOESM1_ESM.pdf]

Namaste suggested interview questions

Intro

Can you tell us about your role?

In your experience, what are the challenges faced by people with advanced dementia with respect to end of life care?

What does good end of life care look like for people with advanced dementia?

Experience of Namaste

What is your involvement with Namaste Care? How did you come to be involved with Namaste?

What appealed to you about Namaste Care?

What was your work like before Namaste?

Can you tell us what kind of Namaste training you've had?

Namaste activities (exploring mechanisms/outcomes)

Talking more specifically about what Namaste involves…

Are there particular activities that are central to providing Namaste, e.g. hand massage; music; scent, etc.?

Is it important that all the activities are incorporated into the programme?

Which are the most beneficial? Why?

What do you think is happening when those activities are done?

What is important about the programme being delivered as a group activity?

For whom and in what circumstances

How do you decide who is suitable for Namaste?

What is the role of the family?

Do you have examples of people it hasn't worked for? Or particular circumstances in which it hasn't worked well?

Why do you think it works / doesn't work?

How do you know it is working? What outcomes have you seen?

Can you recommend other key stakeholders that we might benefit from interviewing?
