# Supplementary material for: Improving living and dying for people with advanced dementia living in care homes: a realist review of Namaste Care and other multisensory interventions
Source: BMC Geriatr. 2018 Dec 6;18:303. doi: 10.1186/s12877-018-0995-9 (PMC6282262; doi:10.1186/s12877-018-0995-9)
Supplement: Supplementary file 2 — Development of Preliminary Programme Theory. (PDF 227 kb) [file 12877_2018_995_MOESM2_ESM.pdf]

Phase 1: Development of preliminary programme theory

If-then statements

Statement
If families participate in Namaste then their experience of visiting improves. They feel supported to engage meaningfully with their relative and are encouraged by the improvements in their quality of life. This will result in family members developing a more collaborative relationship with care home staff.
If Namaste is delivered with intentional presence and through meaningful activities that adhere to the key principles of loving touch and a special environment, then residents feel valued and cared for. Moments of connection will be created between carers and residents – such as attempts to reach out, reciprocate touch or make eye contact – that reflect a sense of 'being with' rather than 'doing for' the person with advanced dementia.
If food and drink that is known to be liked by residents is offered with a Namaste approach, then it becomes a meaningful and pleasurable experience rather than a task; focusing on the senses and becoming another way to make a connection with the resident.
If staff are already attuned to what is needed to provide person-centred care but recognise that they need more skills in advanced dementia care then, with skilled facilitation and training, they will value the opportunity to focus on the emotional aspect of caring and engage fully with the programme. Staff will deliver Namaste with intentional presence.
If Namaste becomes embedded in the care home and is engaged with by everyone, then staff, by reflecting on their everyday interactions with residents, will enact more person-centred care and focusing on the dignity of the resident.

If Namaste is implemented through skilled (external?) facilitation / training with ongoing support from someone with experience of person-centred dementia care, then staff are supported to ‘see the person’, enabling them to move from viewing interactions with residents as task-based to providing *all* care with a Namaste approach.
If a resident’s 5 senses are attended to through a Namaste programme that encompasses all ‘essential’ activities (incl. comfortable room, music, scent, meaningful touch-based.
If staff are already attuned to what is needed to provide person-centred care but recognise that they need more skills in advanced dementia care then, with skilled facilitation and training, they will value the opportunity to focus on the emotional aspect of caring and engage fully with the programme. Staff will deliver Namaste with intentional presence.
If Namaste is delivered in a specially devised environment, then staff are given the time and resources to create a ‘sacred space’ in which to devote themselves to residents, which is distinct from the pressured / busy environment in which they are practicing outside of the Namaste room.
If Namaste is implemented as a structured programme that happens 7 days a week and is planned in collaboration with management and staff, then staff feel empowered to engage with it as a necessary and beneficial part of care for residents who are no longer able to talk.
If Namaste is delivered as a structured programme with a beginning and an end, where residents are welcomed into the specially designed room as part of a group activity, then residents gain a sense of a shared experience and experience reduced isolation and anxiety.
If the same Namaste carer delivers the intervention every day, then residents feel comforted by the familiarity and a bond develops between carers and residents that impacts on wider caring practices. Staff gain in confidence in caring for people with advanced dementia.

If a palliative care approach underpins the Namaste intervention (evidenced by the programme being introduced as something for residents who are approaching the end of life), then staff and families will discuss planning for end of life, what they think their relative may want and the different ways that people living with dementia may die. Family and staff will come to a shared understanding of how to support the resident in different situations that may arise at the end of life.

Preliminary Context-mechanism-outcome configurations with supporting evidence from stakeholder interviews

**Context**	**+**	**Mechanism**	**=**	**Outcome**
How Namaste is introduced to the CH<br>Namaste is introduced to the CH as a structured intervention that happens for 2 hours, twice a day, 7 days a week.<br><br>Staff are given time and resources to make Namaste a specialist intervention.<br><br>There is skilled facilitation in place with ongoing support provided for staff.<br><br>Consideration is given to resourcing the intervention, e.g. creating the special environment ; allocating staff time		Staff feel empowered to engage with it as a necessary and beneficial part of care for residents who do not benefit from other activities.<br><br>Namaste gains credibility as an intervention (both within and beyond the CH).<br><br>Namaste becomes embedded in the CH.		A culture of care develops that embodies Namaste values<br><br>Namaste is sustainable over the long-term
**Evidence in interviews (+ workshop)**				
'When you see proper results is when it's a programme, when it happens 7 days a week and before and after lunch and that involves a huge change in the culture of the care home' Nam03				

‘I think that the benefit is if it’s being presented as a programme and a care programme that unqualified staff can deliver, so it comes with a structure around these very human, very person-centred, very, uh, subjective things that you can do to engage with somebody that I would say good dementia care practitioners would do anyway, but it gives a sense of structure and permission for care staff to do this.’ Nam05

‘I think that it’s very difficult for care home staff particularly or ward staff to, for it to be recognised and something like this that is not a specific task or a procedure, to be given space and time and energy and permission to do.’ Nam05

‘But I think the skill in actually developing somebody’s understanding and application of Namaste is to take them down that journey, to help them connect and emotionally connect and feel if they haven’t done so already…And that’s where the skilled facilitation comes in.’ Nam05

‘I think that’s a really big thing that Namaste adds is that there’s like structured time to really pay attention to the residents and yeah, and give them that extra time, extra, and also the opportunity to make contact with the residents.’ Nam09

‘I think it’s really important for Namaste in the long run that there is someone to like sort of supervise or, you know, just ask them a couple of sort of questions “are you really doing [Namaste activities]?” Nam09

**Context**	**+**	**Mechanism**	**=**	**Outcome**
<u>The characteristics/approach of CH staff</u><br>Staff are attuned to what is needed to provide PCC.<br><br>Staff know their residents well / have engaged in biography work with residents.		Staff reflect on their everyday interactions with residents.<br><br>Staff focus on the dignity of the resident.<br><br>Resident reactions give staff the reinforcement to repeat the approach in their wider practice.		Staff become skilled in providing advanced dementia care that is person-centred.<br><br>Staff have improved assessment of dementia-related symptoms & increased confidence in symptom management

<table>
<tr><td></td><td></td><td></td><td></td><td>Staff have increased responsiveness to distress in residents

Residents become calmer, more relaxed and less distressed

Staff have improved job satisfaction.</td></tr>
<tr><td colspan="5">Evidence in interviews (+ workshop)</td></tr>
<tr><td colspan="5">‘[We’re] actually taking a step back and looking at it from a different point of view [for example] they do need two carers to use a hoist but they don’t need two carers to get washed and dressed, so, and that’s sort of come from this Namaste feeling where we’re trying to protect people’s dignity and let them trust us.’ Nam01

‘I think watching staff I think what you see is that they realise that this person that may be End of Life, they may have really quite advanced dementia but we’re still reaching them… they’re still living, they’ve still got all the things that we have, we just need to find it in a different way and I think that changed people’s perceptions’ Nam01

‘You go in there and you just see residents that won’t, you won’t ever engage with as such and they just kind of really come out of their shell and it is really satisfying after.’ Nam02

‘If I really had to say what I thought the main impact was, I think it’s the impact on the staff because we have our session and my residents go away and it’s gone but I have not lost the sense that actually, I can make you smile, I can do it and also that if I wait you may talk to me and once that gets into my head, why wouldn’t I use that when I’m trying to get you up and dressed or give you a bath or whatever else’ Nam03</td></tr>
<tr><td>Context</td><td>+</td><td>Mechanism</td><td>=</td><td>Outcome</td></tr>
</table>

Characteristics of the Namaste programme / how Namaste is delivered<br>The programme embraces all core elements (as identified in the workshop) and is delivered every day, for 2 hours twice a day.<br><br>Activities are meaningful and attend to all 5 senses.<br><br>Activities are performed with loving touch and intentional presence.<br><br>Food and drink are offered as a social and experiential activity.<br><br>Activities can be adapted to individual circumstances.<br><br>Families and friends participate in the intervention.		The Namaste room creates a 'sacred space' in which the resident is given focused care.<br><br>The slower pace at which Namaste is delivered provides an opportunity to give the resident focused attention.<br><br>Residents feel valued<br><br>Residents feel less isolated<br><br>Relatives and friends feel they are doing something meaningful and positive.<br><br>Families have a better appreciation of the relationship between carers and residents.		Moments of connection occur between residents and staff / family & friends, evidenced by residents' responses e.g. smiling; speech; eye contact; interest in surroundings; reaching/gripping.<br><br>Staff have an increased awareness of residents' needs<br><br>Residents and carers develop a closer bond over time, which impacts on wider caring practices.<br><br>There is better communication between CH staff and the families.<br><br>Improved quality of visits by relatives & friends
**Evidence in interviews (+ workshop)**				

‘We changed our table… so all the residents can sit together with carers next to them to eat their meals, and a lot of that has come from feedback from the staff delivering Namaste that we have such, this close bond and we’re doing this all day and then we’re going into a dining room and separating people and we’re, so this is actually, it’s made the lounge closer I think.’ Nam01

‘Our residents are calmer and eating more, we’ve got residents talking that never used to talk, we’ve got really anxious residents that would be constantly calling for somebody that will actually sit in there for an hour and not call out once…’ Nam01

‘[We] make them feel safe, make them feel comfortable, and then you see the results that we’re getting and I do think that’s the whole package, that’s the taste, that’s the smells, that’s the touch, that’s all of it .’ Nam01

‘it’s a lot of one-on-one time with the residents as well as in the groups, it’s a hell of a lot because I suppose when you’re doing just the care, you’re kind of thinking about all the other things of getting people up and medication and food whereas with that, you just get to kind of focus and bond with the residents.’ Nam02

‘We’re a lot more involved with the residents and it’s a lot more, you can have a lot more conversation with them and because I’ve got a lot of residents but they don’t, don’t really talk that often but obviously with the Namaste it’s kind of brought out of a lot of things in people we never saw.’ Nam02

‘we’ve had a lot of residents upstairs that are quite vocal and can be slightly aggressive during personal care and then that’s kind of improved over time with the Namaste and the whole calming atmosphere they’re not shouting out as much’ Nam02

‘I think when Namaste really works is when you can create a space where people who are overstimulated will settle down, calm down and may go to sleep and that’s great but where people who are withdrawn can actually come out of that shell and can connect and make eye contact and maybe start to try and talk again’ Nam03

‘Very often I think relatives don’t see the positive relationship that carers have because when you’re visiting somebody the staff step back, it’s a visit and they just turn up and say, we need to change your mum or it’s lunchtime, will you feed her or shall I or it’s time we changed her for bed or whatever and so it’s very tasky but in Namaste the family actually see the efforts to get somebody to respond and the positive stuff.’ Nam03
