# Supplementary material for: Improving living and dying for people with advanced dementia living in care homes: a realist review of Namaste Care and other multisensory interventions
Source: BMC Geriatr. 2018 Dec 6;18:303. doi: 10.1186/s12877-018-0995-9 (PMC6282262; doi:10.1186/s12877-018-0995-9)
Supplement: Supplementary file 3 — Table of included Namaste Care studies. (PDF 271 kb) [file 12877_2018_995_MOESM3_ESM.pdf]

Table of included studies that focus on Namaste Care.

All but Magee were included in the scoping in Phase 1.

Author, Year and Country	Type of item (e.g. qualitative, descriptive etc)	Participants (number of severity of dementia)	Core components and duration/frequency	Outcomes
Baldwin (1)<br><br>Australia	Commentary on Chang pilot study of NAMASTE (Nicholls 2013)	NA – describes NAMASTE and talks about Chang study but no participants involved	NA	NA
Duffin 2012 (2)<br><br>UK	Description of implementation of NAMASTE	Home had 33 residents with moderate to advanced dementia and 18 with complex physical conditions. Not clear how many involved in NAMASTE	• Residents spend an hour in the NAMASTE room in the morning and there are elements of the programme in the afternoon.<br>Includes:<br>• Massage<br>• Foot washing<br>• Movement exercises<br>• Watching a DVD<br>• Reminiscence activities	**Anecdotal evidence only (no figures to support the claims)**<br>• staff worked better as a team<br>• Residents sleep better<br>• Less antipsychotic medication<br>• Reduction in falls<br>• Namaste techniques appear to be staff intensive, & there is start-up cost of about £300
Fullarton & Volicer (2013). (3)<br><br>UK	Letter to editor.	9 residents in 1 nursing home. Average age 85. With advanced dementia (other details not given)	• 9AM to 4PM every day<br><br>Activities included:<br>• Massage<br>• Shaving for men<br>• Drinks provided<br>• Sensory activities<br>• Taken outside to feed the chickens	**Presents outcomes but not clear how data was collected**<br>• Decrease in use of psychotics<br>• Apparent improvements in aspects of QoL<br>• Increase in use of analgesics

Author, Year and Country	Type of item (e.g. qualitative, descriptive etc)	Participants (number of severity of dementia)	Core components and duration/frequency	Outcomes
Goodwin 2010 (4)<br><br>Australia	Case report – relates to Chang pilot study of NAMASTE (Nicholls 2013)	Briefly mentions one resident with advanced dementia but little information provided	NA	NA
Kaldy 2008 (5)<br><br>USA	Interview with Joyce Simard – **no data is provided**	JS says Namaste room isn't right for every person with dementia. "You can't have someone who is crying, screaming, or acting out," she said. It also is not a good match for individuals with terminal illnesses—such as cancer—who are still alert and cognitively intact. However, anyone can receive Namaste services in their rooms or after hours with	• Specially designated room<br>• Loving touch<br>• Soft music<br>• Lavender<br>• Stuffed animals<br>• Manicures or hand massages etc	Anecdotal – no evidence reported
King 2013 (6)<br><br>USA	Describes the process of setting up NAMASTE	• People with advanced dementia MMSE 0-7<br>• Participants had challenges in communicating, needed total assistance with personal care and were nonambulatory	• From 3-8pm every day<br>• Small group programs<br>• Sensory programming<br>• Developed a core group of caretakers – because relationships key in building trust<br>• Massage, use of essential oils<br>• Tasty snacks<br>• Cuddly animals and dolls	**Anecdotal evidence** from staff and families about improvements – e.g. improved communication, peace

Author, Year and Country	Type of item (e.g. qualitative, descriptive etc)	Participants (number of severity of dementia)	Core components and duration/frequency	Outcomes
Lourde, K. (2007). (6)<br><br>USA	Commentary on Namaste in 3 nursing homes in the US		• Specially designated room<br>• Loving touch<br>• Soft music<br>• Lavender<br>• Stuffed animals<br>• Manicures or hand massages etc	• Anecdotal evidence about the benefits – e.g. increased family satisfaction, improved care, economic benefits for care homes as increase in referrals
Magee 2017 (7)<br><br>UK (Northern Ireland)	Before/after study looking at feasibility of introducing NC into nursing home and integrating it into usual care	N=9; 1 male, 8 females. 7 diagnosed with dementia, 1 described as having advanced dementia	• Dimmed lighting<br>• Soft background music<br>• Relaxing and calming ambience<br>• Loving touch<br>• Welcome<br>• Snacks and drinks<br>• Aromas<br>• Visual aspects (e.g. lava lamp, pictures)<br><br>Programme ran for 4 weeks.<br>2 sessions a day was soon found to be too much for the staff to engage with, and the programme was reduced to 1 session held after lunch.<br>Run from Monday to Friday (not 7 days)<br>Set up each day took longer than expected which reduced the time for activities	• Small increase in mean weight was recorded over the course of the study (qualitative evidence that residents ate and drank more)<br>• Those with behavioural disturbances showed an improvement (measured using CMAI, CBS, Cornell)<br>• Suggest that grouping together residents with similar interests would make tailoring the activities easier.

Author, Year and Country	Type of item (e.g. qualitative, descriptive etc.)	Participants (number of severity of dementia)	Core components and duration/frequency	Outcomes
McCormick 2011 (8)<br><br>USA	Describes use of Namaste by EPOCH, Massachusetts (one of several papers about EPOCH)			Anecdotal
Manzar, B., & Volicer, L. (2015). (9)<br><br>UK	Before/after pilot study & qualitative study. 9 residents, 9 relatives, 8 staff (6 hands on Namaste carers & 2 senior staff).	N=9: 1 resident was moderately impaired, 4 residents were severely impaired and 4 were very severely impaired	• Takes place every day for 4 hours, 2 hours in morning and 2 in afternoon<br>• Specially designated room<br>• Food and drink treats<br>• Lavender oil<br>• Reminiscence<br>• Stuffed animals and dolls	Data collected at baseline and 3 and 7 weeks by carer.<br>Pain (PAINAD)<br>QoL (QUALID) – say it improved QoL score in all and decreased perception of pain in some residents
McNiel, P., & Westphal, J. (2016). (10)<br><br>USA	Qualitative study. 14 staff members (certified nursing assistants, registered nurses, clergy, and therapists). 1 long-term care facility in the US.	Eligibility to participate in the Namaste Care™ program included diagnoses of Alzheimer's disease, dementia, strokes, cognitive and behavioural issues. Staff were encouraged to use nursing judgment and invite residents to trial the Namaste Care™ program for a brief period of time to assess potential resident benefits for program enrolment	• Maximum capacity 8<br>• Little description of what components of NAMASTE they used	**Anecdotal evidence** from staff and families about improvements

Nicholls et al (2013). (11)<br><br>Australia<br><br>NB this study by is referred to in several publications – but we were unable to find a publication reporting final results	Qualitative<br>7 focus groups consisting of 31 participants were conducted separately for each of the study cohorts: family members of residents, AINs and RNs.<br>6 RACFs (3 intervention, 3 control)	Mini-Mental State Examination score < 7 and bed-fast or chair-fast –<br>End stage of dementia trajectory, requiring palliative care<br><br>but this paper only reports staff and relatives' views of the impacts of NAMASTE	High touch intervention – combines 1) intensive train the trainer package for care staff; 2) family conferences facilitating end of life discussions, 3) delivering NAMASTE Care Programme (not further defined)	• Qualitative exploration of importance of touch
Simard 2005 (12)<br><br>USA	Describes set up of Namaste in care home in Vermont. Focuses on one resident	One man with advanced dementia at end of life	• Usual elements of the program but not clear if delivered in group or one to one	• Anecdotal evidence that quality of death for this resident was improved
Simard 2007 (13)<br><br>USA	Describes the use of Namaste by one long-term care company (EPOCH in Massachusetts)	Suggests participants should have: 1. a diagnosis of irreversible dementia, 2. an MMSE score <7, 3. unable to participate in scheduled activities, 4. non-ambulatory, 5. difficulties communicating, 6. total care with ADLs. Others recommended to benefit from Namaste are people with COPD, Parkinsons disease and other terminal illnesses or those who are agitated	Activities include:<br>• Aromatherapy diffuser<br>• Essential oils, especially lavender<br>• Stuffed animals<br>• Music<br>• Sensory material<br>• Humorous items (e.g. wigs)<br>• Antique items<br>• Reading material	• Anecdotal evidence about benefits for staff and residents

Author, Year and Country	Type of item (e.g. qualitative, descriptive etc.)	Participants (number of severity of dementia)	• Core components and duration/frequency	Outcomes
Simard & Volicer (2010). (14)<br><br>USA	Before/after study. 86 residents. 6 EPOCH Senior Living Healthcare Centres in the USA.	N=86: 1 participant was borderline intact, 2 had mild CI, 23 had moderate CI, 23 had moderately severe impairment, 14 had severe impairment, and 23 had very severe impairment. However, none of the residents was rated comatose.	• Namaste carers were selected on desire to be involved in the program.<br>• The number of residents in the program ranged from 6 to 11 with 1 Namaste carer or another staff person always present in the room.<br>• The program was supervised usually by a Director of Nursing or Assistant Director of Nursing	Analysis of minimum data set when residents had been involved in programme for at least 30 days showed decrease in resident's withdrawal, Social interaction & delirium indicators
Simard 2012 (15)	Describes NAMASTE and one of the first residents to be part of the programme	Simard says she uses this story (of Evelyn Groves) to encourage carers to try Namaste on even the most difficult residents	• Specially designated room<br>• Loving touch<br>• Soft music<br>• Lavender<br>• Stuffed animals<br>• Manicures or hand massages etc	Anecdotal – describes benefit for one resident
Soliman & Hirst (2015). (16)<br><br>UK	Before and after study.<br>2 care homes in London.	N= 11-14 people with advanced dementia	• 3-5 sessions in each home per week<br>activities tailored to individual preferences, including:<br>• Hand/foot massage<br>• Reminiscence;<br>• Food and drink treats;<br>• Personal care including hair brushing or face washing<br>• Specially designated room	Outcomes included:<br>• Aggressive or challenging behaviour (10/14 showed a reduction)<br>• QoL – improvements shown<br>• Sleeping patterns<br>• Appetite<br>• Staff, residents and relatives' satisfaction – data not available

Author, Year and Country	Type of item (e.g. qualitative, descriptive etc.)	Participants (number of severity of dementia)	• Core components and duration/frequency	• Outcomes
Stacpoole 2014 (17)<br><br>UK	Before/after & qualitative evaluation (staff / relative separate focus groups and manager interviews). Action research. 30 Residents and also relatives, care staff and managers. 5 care homes in England.	30 residents with a dementia diagnosis and a Bedford Alzheimer's Severity Scale score of >16	• Takes place every day for 4 hours, 2 hours in morning and 2 in afternoon<br>• Dedicated space<br>• Soft music, scents, greenery<br>• Residents are welcomed by name<br>• Pain management<br>• Sensory stimulation<br>• Food treats/hydration etc	• Primary measures NPI-NH and Doloplus-2 behavioural pain assessment<br>• Baseline and at 3, 1-2 month intervals<br>• neuropsychiatric symptom severity & disruptiveness decreased in four CHs but increased in one CH<br>• Slight reduction in effectiveness towards end of intervention
St. John & Koffman 2015 (18)<br><br>UK	Qualitative - feasibility and effectiveness of Namaste Care in a large inner-city teaching hospital in UK	8 semi-structured, face-to-face interviews with members of the multidisciplinary ward team<br><br>No residents involved in this study. Type of participants not specified	• Group or one to one sessions (Mon-Fri). Grp sessions last 1 hr and one to one between 20-30 minutes – but doesn't say how often each person receives it<br>• Units day room developed into a sensory room<br>• Loving touch, foot and hand massage, reminiscence etc	• Anecdotal, qualitative reports from staff on the potential benefits.
Trueland 2012 (19) UK (relates to Stacpoole 2014)	Commentary – describing NAMASTE and the Stacpoole study	Not specified but assume is same as Stacpoole 2014	• Residents are welcomed by name<br>• Settled comfortably with blankets and pillows<br>• Soft music<br>• Massage<br>• Reminiscence<br>• Delicacies such as ice lollies and orange slices	**Anecdotal**<br>• Better pain assessment<br>• Reduction in pressure ulcers<br>• Increased intake of fluids

References

1. Baldwin J. Honouring the spirit within. Aust J Dement Care. 2012;1(3):19.
2. Duffin C. How namaste principles improve residents' lives. Nurs Older People [Internet]. 2012 Jul [cited 2017 Jan 16];24(6):14–7. Available from: http://www.ncbi.nlm.nih.gov/pubmed/22900391
3. Fullarton J, Volicer L. Reductions of antipsychotic and hypnotic medications in Namaste Care. J Am Med Dir Assoc [Internet]. 2013 Sep [cited 2017 Jan 16];14(9):708–9. Available from: http://linkinghub.elsevier.com/retrieve/pii/S1525861013003459
4. Goodwin L. Touch their soul and spirit. Blacktown Advocate. 2010;7.
5. Kaldy J. "Namaste" care honors the spirit within advanced-dementia patients. Caring Ages. Baltimore, Maryland: Lippincott Williams & Wilkins; 2008;9(4):26.
6. Lourde K. Namaste: Honoring the spirit within. Long-Term Living. 2013;62(2):14–15,23.
7. Magee M, Mccorkell G, Guille S, Coates V. Feasibility of the Namaste Care Programme to enhance care for those with advanced dementia. Int J Palliat Care Nurs. 2017;23(8):368–76.
8. Mccormick C. Namaste Care at EPOCH Senior Living. cmccormick@capecodonline.com. 2011;
9. Manzar B, Volicer L. Effects of Namaste Care: Pilot Study. Am J Alzheimer's Dis. 2015;2(1):24–37.
10. McNiel P, Westphal J. Namaste Care : A Person-Centered Care Approach for Alzheimers and Advanced Dementia. West J Nurs Res. 2016 Nov 24;
11. Nicholls D, Chang E, Johnson A, Edenborough M. Touch, the essence of caring for people with end-stage dementia: A mental health perspective in Namaste Care. Aging Ment Health. 2013 Jul;17(5):571–8.
12. Simard J. Namaste, giving life to the end of life. Alzheimers Care Q. 2005;6(1):14–9.
13. Simard J. Silent and invisible; nursing home residents with advanced dementia. J Nutr Health Aging [Internet]. 2007 [cited 2017 Jan 16];11(6):484–8. Available from: http://www.ncbi.nlm.nih.gov/pubmed/17985064
14. Simard J, Volicer L. Effects of Namaste Care on Residents Who Do Not Benefit From Usual Activities. Am J Alzheimer's Dis Other Dementias®. 2010 Feb 1;25(1):46–50.
15. Simard J. One small miracle. J Gerontol Nurs. 2012;38(9):54–6.
16. Soliman A, Hirst S. Using sensory activities to improve dementia care. Nurs Times. 2015;111(27):12–5.
17. Stacpoole M, Hockley J, Thompsell A, Simard J, Volicer L. The Namaste Care programme can reduce behavioural symptoms in care home residents with advanced dementia. Int J Geriatr Psychiatry [Internet]. 2015 Jul [cited 2017 Jan 16];30(7):702–9. Available from: http://doi.wiley.com/10.1002/gps.4211
18. John KS, Koffman J. Acceptability of Namaste Care for patients with advanced dementia being cared for in an acute hospital setting. 2015;1–13.
19. Trueland J. Soothing the senses. Nurs Stand. 2012;43:20–2.
