# Supplementary material for: Improving living and dying for people with advanced dementia living in care homes: a realist review of Namaste Care and other multisensory interventions
Source: BMC Geriatr. 2018 Dec 6;18:303. doi: 10.1186/s12877-018-0995-9 (PMC6282262; doi:10.1186/s12877-018-0995-9)
Supplement: Supplementary file 4 — Table of included primary studies Phase 2. (PDF 277 kb) [file 12877_2018_995_MOESM4_ESM.pdf]

Table of Included studies - Primary studies

Author and year	Country	Study type	Study focus	Intervention	Duration & intensity	Delivered by	Setting	Sample size
Anderson 2011	Australia	within subject controlled study	Multisensory	Snoezelen room. 14 staff members received enhanced snoezelen training	1x week for 6 weeks. Staff encouraged to continue each session for 20 minutes unless the resident appeared distressed.	Care home staff	Snoezelen room	12
Behrman 2014	UK	Discussion paper	Multisensory	NA - discussion on senses in management of dementia	NA	NA	NA	NA
Belgrave 2009	US	Before/after	Multisensory, Music	All groups received music therapy provided by a music therapist who sang and played the guitar. Two groups also received touch as part of the intervention (either expressive or instrumental touch).	9x 30 min sessions each over 2 weeks (each condition 3 times each)	Music therapist	At bedside or in private room	9
Bergland 2015	Norway	Qualitative	Environment	NA - looks at caregivers perceptions of mealtime agitation (for example impact of social interaction and stimuli)	NA	NA	Dining area	21
Burns 2011	UK	RCT	Aromatherapy	Participants were allocated to 1 of 3 groups: placebo medication and active aromatherapy; active medication and placebo aromatherapy; or placebo of both.	4 weeks	family carers	Not specified	114 (81 completed the 12 wk assessment)
Cameron 2011	UK	RCT	Aromatherapy	2% lemon balm oil rubbed into the forearm for one minute twice a day.	1 minute of massage 2 x day for 3 weeks.	Nurses	CH	18
Chenoweth 2009	Australia	RCT	Other	3 groups: person-centred care, dementia-care mapping, or usual care. Carers received training and support in either intervention.	4 months.<br>Mapping was 6h per day for 2 days	Researchers	Around the CH.	236 (of 289 recruited) residents were randomly assigned. (109 mapping group; 98 PCC group; 82 control)

Table of Included studies - Primary studies

Chu 2014	Taiwan	RCT	Music	Music therapy which included gross & fine motor movements performed to music; rhythm playing along with music; listening to popular music; rhythm playing with instrumental accompaniment; and singing with instrumental accompaniment.	12 sessions of group music therapy (two x 30-min sessions a week for 6 weeks)	Trained music therapist with experience in dementia	The recreation rooms of each facility.	100 (49 intervention, 51 control)
Cohen-Mansfield 2011	US	Observational	Environment, Multisensory, Music,	Twenty-five predetermined engagement stimuli were presented to participants over a three-week period (approximately 4 stimuli per day) categorised as; live human social stimuli, simulated social stimuli, inanimate social stimuli, reading stimulus, manipulative stimuli, a music stimulus, task and work-related stimuli, and two different personalized stimuli, based on the study participant's self-identity.	Engagement trials took place between 9:30 am – 12:30 pm and between 2 pm – 5:30 pm, approximately four activities each day for three weeks. (duration of each activity not stated).	Research assistants	Not stated, but description suggests a communal area	193
Cohen-Mansfield 2012 - same data set as Cohen-Mansfield 2011	US	Observational	Environment	See Cohen-Mansfield 2011	See Cohen-Mansfield 2011	See Cohen-Mansfield 2011	See Cohen-Mansfield 2011	193
Cohen-Mansfield, 2012 (same data set as Cohen-Mansfield 2011)	US	Observational	Multisensory	Participants were presented with 25 predetermined stimuli. Categories as before	A three-week period (approximately 4 stimuli per day) between 9:30 - 12:30 pm and between 2 pm-5:30 pm	Researcher	Actvity or dining room	193

Table of Included studies - Primary studies

Cohen-Mansfield, (2011) (same dataset as Cohen-Mansield 2012)	US	Observational	Multisensory	Each participant was presented with 25 predetermined engagement stimuli in random order. Categories as before	3 weeks, 4 stimuli per day. Between 9:30 -12:30 pm and between 2 pm-5:30 pm	Researcher	Activity or dining room	193
Cohen-Mansfield 2010	US	Observational	Other	Same as 2011 and 2012	Same as 2011 and 2012	Researcher	Activity or dining room	193
Cohen-Mansfield 2016	US	Observational	Environment, Other, Structure	Looks at unmet need within context of treatment arm of RCT. Identifies need for sensory stimulation and social interaction.	NA	NA	Around the care home	89
Cohen-Mansfield 2010	US	Observational	Multisensory	See Cohen-Mansfield 2011	See Cohen-Mansfield 2011	Researchers	Nursing home specific space not described	111
Cohen Mansfield 2015	US	Observational	Music, Other, Touch/massage	Non pharmacological interventions - includes sensory stimulation, social interventions (e.g. lifelike doll), music, outside activities and one to one with research assistant. RA collected data on persons needs and preferences from formal and informal caregivers and from direct observations.	5 x week for 2 weeks. Amount and duration of interventions varied	Researcher	Not specified	93
Collier 2008 and collier 2010	UK	RCT	Multisensory	Multisensory - aimed at stimulating all 5 senses.	12 sessions, length not specified	Not clear	This activity was run in a quiet area, quiet room or purpose built MSE.	30 (17 in intervention group and 13 in control)

Table of Included studies - Primary studies

Cruz 2011	Portugal	Before/after	Multisensory	Motor and multisensory stimulation strategies during residents' morning care routines by staff after the provision of group training and individualized assistance. This included things such as warm towels or gentle massage	Staff participants received eight 60-minute training sessions.	Care home staff who were trained by members of MDT	In residents own rooms (as part of washing, dressing etc)	6
Cruz 2013	Portugal	Other	Multisensory	Multisensory and motor based group activity program. Used residents preferences and lifetime histories to frame sessions	1 x week for 16 weeks. Each session about 45 minutes	Facilitators - physical therapist, gerontologist, activities coordinator	Quiet room with proper lighting and without extraneous stimuli (e.g TV from outside)	4
Fu 2013	Australia	RCT	Aromatherapy, Touch/massage	3 groups: Combination (aromatherapy and hand massage ; Aromatherapy ; Placebo control (water spray). Aromatherapy spray was used (allowing the potential confounding effect of massage to be controlled for). Given the likelihood that participants might have compromised olfactory systems a direct spray onto individuals' upper chest was used.	Twice daily at 2 time periods, 9-11 am and 2-4 pm, 7 days a week for 6 weeks. (Hand massages were for 2.5 minutes each hand).	Researchers	A quiet and private environment, such as the participant's room.	61 (combination n = 19 ; aromatherapy n = 22 ; control n = 20)
Fujii 2008	Japan	RCT	Aromatherapy	Lavendar aroma therapy. Two drops of lavandar oil applied to the waist band of the person's underwear	The intervention was given 3x day for 4 weeks	Not stated.	Long term care facility	28

Table of Included studies - Primary studies

Gotell 2009	Sweden	Observational	Music	3 different options were tested: the ''usual'' caring caring routine (i.e., the control situation); a caring routine done with recorded music playing in the background (''back- ground music''); a caring routine in which the caregiver sang to and/or with the patient (''caregiver singing'').	The sessions occurred between 7 am and 9 am and lasted between 6 and 22 minutes.	Care home staff	In a bathroom	14 (9 people with severe dementia & 5 professional caregivers)
Goto (2014)	US	Non randomised controlled	Environment/Multisensory	Snoezelen room OR an indoor Japanese garden	15 minutes, 2x week (3 weeks for Snoezelen room, 4 weeks for Japanese garden)	Researcher	Japanese garden or Snoezelen room	18
Hammar, (2011)	Sweden	Observational	Music	MTC (music therapy caregiving) was implemented during morning care situations while PWD were being cared for. Involved caregivers singing songs.	In all, every PWD participated over a two-month period	Care home staff	Not specified	10
Harris 2012	US	Other	Touch/massage	Slow stroke back massage (SSBM) done at night when resident was ready to go to sleep. SSBM is characterized by long, slow, gliding, repetitive strokes from the sacral to cervical regions of the spine	3-minute SSBM at bedtime for two nights.	Researcher trained in SSBM	participants rooms	40
Hsu 2015	UK	RCT	Music	1:1 live interactive music therapy. Therapists used musical, vocal, bodily and facial expressions. After each session, two video clips were presented to the care staff participants in the intervention group unit, in order to communicate elements of music therapy to carers.	1:1 MT once x week for 5 months. Each session was 30 minutes. On average people missed 5 sessions	Music therapist. Residents received sessions from the same music therapist throughout the project	Quiet room on the unit.	17 care home residents and 10 care home staff

Table of Included studies - Primary studies

John 2015	UK	Qualitative			group sessions last an hour, one to one between 20 and 30 mins. Most have 3 sessions a week	Activity coordinators	Day room which has been developed into a sensory room.	
Kellett 2010	Australia	Qualitative	Other	Qualitative evaluation of The FBW process which involved families and staff completing a set of exercises designed to help them build a biography of the life of the person with dementia. .	6 x week, two-hour sessions	A researcher facilitated	NA qualitative sub-study	14
Kupeli 2016	UK	Qualitative	Other	NA - realist study exploring quality of end of life care for providing good palliative care to people with advanced dementia in care homes	NA	NA	NA	14
Lancioni 2013	Not clear	Non randomised controlled	Music	Sessions involved music stimulation (i.e. active and passive conditions) or no music stimulation. In the active condition, the patients used a simple hand response and a micro switch to self-regulate music stimulation inputs. In the passive condition, music stimulation was automatically presented throughout the sessions.	3-7 x day, duration between 5-10 minutes.	Care home staff	A room of the residential center that the patient attended	6
Lape 2009	US	NA	Environment, Multisensory	Snoezelen room- including fibre optics, mirrors and visual effects, a sound machine, tactile items, scented objects and a glider rocket.	30-45 minutes sessions, 3 x week for 6 weeks.	Researcher	Snoezelen. 9'x9' room.	not reported

Table of Included studies - Primary studies

Litchke 2014	US	Qualitative	Multisensory, Touch/massage	Multisensory chair yoga program - included touch, visual modelling & auditory stimulation.<br>A namaste greeting was used to provide a consistent end to each session and thank them for attending.	2x week for 10 weeks, for 30-55 minutes.	Researcher who is a certified yoga instructor.<br>2 recreaction therapy graduates assisted each session	Not stated	26
Lykkeslet 2014	Norway	Qualitative	Multisensory	Qualitative evaluation of multisensory programme. Includes combination of sensory activities similar to Namaste.	Not clear	Care home staff	The dining room	not reported
Magee (2017).	Northern Ireland	Before/after	Multisensory	Namaste Care - Programme included welcome, hand massage, scent, touch to stimulate pleasant memories, music. Tailored to the needs of individual residents (biographical details obtained from family members). The facilitator (MM) held a training session for the staff and the carers to be involved with the programme prior to its commencement.	2 hours of activities, 5 days a week for 4 weeks	MM (lead author and Palliative Care Nurse Specialist) ran the first session then the activity therapist delivered the rest with help from relatives. Two staff members were allocated to assist in the programm	Residents lounge	9
Manzar 2015	UK	Before/after	Other		every day, 2 hrs before and 2 hrs after lunch	Care home staff	A specific room was selected and furnished in a style that was comfortable and familiar for the individuals.	9

Table of Included studies - Primary studies

Mariko (2015)	Japan	RCT	Touch/massage	Touch therapy in which - touch was applied on the Shinchuu, (between T3 and T4 vertibrae).	2x day (morning and afternoon) for 4 weeks, each instance lasted between two and three minutes.	Caregivers (not specified)	Not stated	21
Maseda 2014	Spain	RCT	Multisensory	Smultisensory environment (snoezelen), individualised activities and control. Data on the participants' sensorial preferences and interests were collected to design the content of the sessions	2 x week for 16 weeks.	All sessions were conducted by professionals (occupational therapist or psychologist) with equivalent education and training in the methodology used.	A multisensory environment (type of room or space not specified)	30
McDermott (2014)	UK	Qualitative	Music	Music therapy where clients were encouraged to explore musical instruments and/or their voice with the therapist. The method of facilitation and clinical intervention varied, and was determined by the clinical needs of each client and were shaped by the theoretical orientations and experiences of the music therapists.	NA (intervention varied)	Music therapists - all had a minimum of four years' experience of working with clients with dementia in residential settings.	Various	53
McNeil 2016	US	Qualitative				Care home staff	Specially designed NC room.	
Milev 2008	Canada	RCT	Environment, Multisensory	Participants were brought to the snoezelen room and exposed to MSS through 1:1 interaction with facilitator.	1-3 x week for 12 weeks. Each session lasted 30 minutes (or less)	Qualified Snoezelen facilitaor	Snoezeln room	18

Table of Included studies - Primary studies

Moyle 2014a (Moyle 2014b is the same study)	Australia	RCT	Touch/massage	Foot massage - light presssure with an unscented lubricant. In quiet presence assistant sat quietly at residents feet for 10 minutes without talking of making physical contact.	10-min foot massage (intervention) or quiet presence (control), every weekday for 3 weeks. Massage group received an average of 12 massages, control group average of 14 QP sessions.	Research assistants who were trained massage therapists	Residents bedroom	55
Moyle 2014b (Moyle 2014a is primary study)	Australia	RCT	Touch/massage	See Moyle 2014a	See Moyle 2014a	Nursing students trained to deliver either foot massage or quiet presence interventions	Residents bedroom	53
Murphy (2017)	UK	Qualitative	Other	NA	NA	NA	NA	50 (30 care staff, 8 family carers, 3 dieticians, 9 speech therapists)
Nair (2011)	Australia	observational with cross over	Music	Baroque music played in communal areas. Residents were free to wander in and out of their rooms during this time.	music intervention - music played for 4 weeks from 3 pm to 7 pm)	CD player in the common area, no person to person aspect to the intervention	communal area	75
Narme 2014	France	RCT	Music	Music played on a CD. Excerpts covered a range of styles and were calming with slow or moderate tempo at the beginning and the end of the session with a higher tempo in the middle of the session. Participants asked to participate by singing or using percussion instrument.	One hour twice a week for 4 weeks. Total of 8 hours. Same play list in same order for every session.	Two facilitators. Neither had any prior education in music therapy	Not stated	intervention music group (n=18), control group (n=19). 48 were originally randomised but only 37 completed the intervention

Table of Included studies - Primary studies

Nicholls 2013	Australia	Qualitative		High-touch intervention based on NS. Involved train the trainer package, family conferences and the NC programme	Not specified	care home staff	Not clear	31 - staff and family members.
Quell 2008	Sweden	Non randomised controlled	Touch/massage	Tactile stimulation (TS) is a form of touch massage aimed at reaching the skin touch receptors. No background music was allowed and noise and interruptions kept to a minimum. TS generally applied to hands, arms, feet, or legs were the areas generally chosen.	a minimum of once a week for 28 weeks with each sequence lasting at least 20 minutes.	Care home staff who received theoretical as well as practical instruction in TS. .	Residents own rooms	20
Raglio 2015	Italy	RCT	Music	Music therapy - during the session, the music therapist built a relationship with the PWD by singing and using melodic and rhythmic instruments (improvisation), facilitating the expression and modulation of the PWD's emotions and promoting "affect attunement" moments<br>Listening to music - therapist created a playlist on basis of interviews with PWD	20 sessions - either individualised music therapy or individualised listening to music. 30 minutes	Music therapist	Quiet medium-sized room	120
Raglio (2010).	Italy	RCT	Music	1 experimental group (MT and standard care) & 1 control group (standard care only).	The experimental group received three cycles of 12 30min MT sessions each, three times a week.	Music therapists	Not reported	51 (of 60 recruited)

Table of Included studies - Primary studies

Rodriguez-Mansilla, (2015)	Spain	Other	Touch/massage	One group received ear acupressure and the other massage.	Duration and frequency of acupuncture not clear. The massage was applied on the lower limbs and back during 20 minutes, on a daily basis from Monday to Friday	The ear acupressure techniques were performed by a qualified acupuncturist . The massages were performed by a qualified physiotherapist	Not reported	111
Sakamoto (2012)	Japan	RCT	Aromatherapy	Lavender vs placebo. The lavender group received continuous olfactory stimulation from a lavender patch. The patch attached to the inside of the clothes near the neck, the odor is faint only the person wearing the patch can smell it. The placebo group received an unscented patch which looked identical.	All day, every day for 12 months.	Nursing home staff	Patch was attached to resident	N=145, intervention n=73, control n=72
Sakamoto 2013	Japan	RCT	Music	Compared a non-intervention Control group, a Passive music intervention group, and an Interactive music intervention group.	Each intervention was performed for 30 min 1 x week (10 sessions in total)	Music facilitators included two music therapists, four OTs, and six nurses with knowledge of severe dementia symptoms. All received training.	A familiar setting for resident	39

Table of Included studies - Primary studies

Sanchez 2016	Spain	RCT	Multisensory	2 groups: MSSE (multisensory stimulation environment) and individualized music sessions.<br>The MSSE group participated in multisensory sessions in a Snoezelen room	x2 30-min weekly sessions over 16 weeks	Professionals in the field of psychology or occupational therapy, with equivalent education and training in the methodology used.	MMSE<br>Snoezeen; music sessions in a quiet room	18 (9 each group) of 22 recruited
Simard 2010	US	Before/after	Namaste Care	Involved: welcome, comfortable seating, pain monitoring, meaningful activities, snacks and drink, touch	7 x week, for approx 5 hours a day.<br>The number of residents in the program ranged from 6 to 11	Namaste Carers (certified nursing assistants).	4 facilities designated a room that was only used for Namaste Care and was decorated with soft colors and had a homey feeling.	86
Soliman 2015	UK	Before/after	Namaste Care	Involved: welcome, comfortable seating, pain monitoring, meaningful activities, snacks and drink, touch	3-5 sessions in each home each week. Some homes offered sessions of 90 minutes others 2 hrs	Care home staff	Namaste Care area (where residents can feel they are entering a special space)	14
Staal, (2007)	US	RCT	Multisensory	Randomized to receive either: multi-sensory behavior therapy (MSBT; one to one individualized sensory stimulation) or a standard structured activity session i.e. one to one attention using therapeutic recreation activities such as play dough.	6 sessions 25- 30 minutes per session	Not clear	Not reported	24

Table of Included studies - Primary studies

Stacpoole 2016	UK	Other	Namaste Care	Involved: welcome, comfortable seating, pain monitoring, meaningful activities, snacks and drink, touch	2x day, 7 days a week	Namate Care workers	Not clear - some homes had special NC space but others did not seem to	37 enrolled (30 completed the study)
Strom 2017	Ireland	RCT	Multisensory, Other	3-groups (Sonas, reading, and control group).<br>The Sonas programme is a multi-sensory stimulation programme. It involves cognitive, sensory, and social stimulation, including all 5 senses. The programme is a therapeutic activity for people who have significant communication impairment, primarily as a result of dementia.	Twice a week for 24-weeks. Each session lasted 45 mins.	A person trained in the Sonas programme (Sonas licensed practitioner) and an assistant.	Not reported	105 of 120 recruited: Sonas group (n = 45), the reading group (n = 27) or the control group (n = 33)
Sung 2010	Taiwan	Before/after	Music	The preferred music listening intervention was based on an individualized music protocol.<br>The control group had usual care	30 mins mid afternoon, 2x week for 6 weeks. The participants listened to their preferred music via CD players.	Trained nursing staff	A familiar setting	52 (29 intervention & 23 control)
Sung 2012	Taiwan	RCT	Music	Music intervention using percussion instruments with familiar music	12 sessions of group music intervention over 6 weeks lasting for 30 minutes	Research assistants trained in music intervention	Residential care facility	55 (27 in experimental group and 28 in control)
Suzuki 2010		Non randomised controlled	Touch/massage	The intervention was a Swedish complementary care method called tactile massage.	Participants received tactile massage 5 times a week for 6 weeks for a total of 30 times. Each session took place at 4-5pm and lasted approx. 30 minutes.	Ward nurses and collaborating nurse researchers who had participated in a 2 day training course	not specified	40 (20 people in the intervention group and 20 people in the control group).
Tuckett 2015	Australia	Qualitative	Music	Music therapy	2x week for 12 weeks, 45-60 mins each	Music therapist	In the dementia-specific unit	30

Table of Included studies - Primary studies

Van Vracem 2016	Belgium	Qualitative	Multisensory, Other	NA: Created a framework with seven types of environmental interventions for agitation in dementia	NA	NA	NA expert panels	27
Vezina 2011	Canada	Qualitative	Identity cues	NA - focuses on role of life story/biography	NA qualitative	NA qualitative	NA qualitative	21
Vink 2013	The Netherland	RCT	Music	Residents participated in group interventions (music therapy or general activities) with a maximum of five residents. In music therapy condition, sessions started with a welcome song after which residents listened to music selected, sung or played by the therapist. If possible they actively participated in music activities. Therapist adjusted the level of each intervention to each individual's capacities.	Twice weekly for 4 months. All sessions were 40 minutes. (maximum of 34 sessions each resident / TOTAL: 235 music therapy and 180 general activities)	A music therapist with at least 5 years working experience. The recreational activities were provided by occupational therapists.	Both interventions were provided in separate rooms, away from the ward.	77 residents (43 intervention and 34 control)
Ward-Smith 2009	US	Restrospective study	Multisensory	Multisensory stimulation environments The interventions included in this study were: music, light, touch, message, and aromatherapies.	Each treatment is provided over a 15- to 20-minute period, between 1300 and 1600. A total of 84 MSE-based interventions were provided.	session coordinated by activity director	Multisensory simulation room (MSSR)	14 (7 intervention, 7 control)
