# Supplementary material for: Improving living and dying for people with advanced dementia living in care homes: a realist review of Namaste Care and other multisensory interventions
Source: BMC Geriatr. 2018 Dec 6;18:303. doi: 10.1186/s12877-018-0995-9 (PMC6282262; doi:10.1186/s12877-018-0995-9)
Supplement: Supplementary file 5 — Table of included reviews Phase 2. (PDF 205 kb) [file 12877_2018_995_MOESM5_ESM.pdf]

Table of Included studies - Systematic Reviews

Author and year	Aim/purpose	Study focus	included studi	ype of participan	Main findings
Chang 2015	Investigate efficacy of music therapy on disruptive behaviours, anxiety levels, depressive moods and cognitive functioning in people with dementia; and clarify which interventions, therapists and participant characteristics exerted higher and more prominent effects	Music	10 RCTs	PLWD (5 of the 10 included studies included people with severe dementia)	Music therapy significantly improved disruptive behaviours and anxiety levels and might affect depressive moods and cognitive functioning. Sessions twice a week (inclusive) exerted a larger and more significant effect on disruptive behaviours, anxiety levels, and depressive moods than did weekly sessions. Music therapy more effective in people with mild to moderate dementia than those with moderate to severe dementia.
Chatterton 2010	To illuminate who sings to PWDs, and with what objectives and effects, to address the question of whether it is the singer or the singing which is effective.	Music	16	PLWD	Evidence that individual singing can be effective depending on context and goals. Authors suggest music therapists should empower professional caregivers to sing sensitively to PWD during caregiving activities
Clare 2010	An integration of the available evidence regarding 3 levels of complex awareness ('on-line monitoring', 'evaluative judgement' and 'meta-representation') and fundamental awareness of sensory and perceptual stimuli, in moderate to severe dementia.	Awareness	13	PLWD (moderate to severe)	Sensory and perceptual awareness can be detected even in people with very severe or end-stage dementia, while some aspects of complex awareness (categorized into 3 types *see below) may be retained into the severe stages.
Conn 2010	Highlights some important advances in pharmacological and nonpharmacological interventions	Mixed	Not clear	Older adults with psychiatric disorders	There is some evidence to support the use of psychosocial interventions for behavioral symptoms, including those utilizing sensory interventions such as aromatherapy and calming music; cognitive stimulation; and staff training. There is also some evidence to support the use of psychotherapies such as reminiscence therapy for depression.

Enmarker 2010	to describe, from a nursing perspective, aggressive and violent behaviour in people with dementia living in nursing home units and to find alternative approaches to the management of dementia related aggression as a substitute to physical and chemical restraints.	Mixed	21	PLWD	Supports importance of social interaction in person centred approach. Residents may experience isolation and loneliness
Ennis 2013	The review focuses on the role of spiritual nursing interventions in the improved health outcomes of older adults with dementia	Other	8	PLWD - 1/8 specifies severe dementia	Research indicates that the memory needed to explore one's spirituality may be spared the effects of dementia. This preservation of memory allows older adults with dementia to benefit from spiritual nursing interventions, especially music and rituals.
Forrester 2014	To assess the efficacy of aromatherapy as an intervention for people with dementia.	Aromatherapy	7 (but data only used from 2)	PLWD	benefits of aromatherapy are not clear from current evidence
Guetin 2013	To present the developments of music therapy in France, its techniques, mechanisms and principal indications, mainly in the context of Alzheimer's disease	Music	11 RCTS	PLWD	Provides some evidence for the benefits of music therapy

Table of Included studies - Systematic Reviews

Kim 2014	To assess the effectiveness of occupational therapy on behavioural problems and depression in patients with dementia.	Other	9 RCTs	PLWD	Sensory stimulation occupational therapy was associated with a statistically significant improvement (small effect) in behavioural problems compared with usual care Environmental modification occupational therapies had no significant effect on behavioural problems (two RCTs). Functional task activity occupational therapies had no significant effect on depression (three RCTs). No trials were identified that assessed the remaining combinations of occupational therapy classification and outcome type (behavioural problems or depression).
Livingston 2014	Review & synthesise evidence for effectiveness of non-pharmacological interventions for reducing agitation in dementia,	Other	160 studies	PLWD	Person-centred care, communication skills and DCM (all with supervision), sensory therapy activities, and structured music therapies reduce agitation in care-home dementia residents
McDermott 2013	Provide insight into the possible mechanisms of actions of music therapy.	Music	18	PLWD	Evidence for short-term improvement in mood and reduction in behavioural disturbance was consistent, but there were no high-quality longitudinal studies that demonstrated long-term benefits of music therapy.
Nguyen (2008)	We aimed to review the evidence supporting the use of aromatherapy in BPSD	Aromatherapy	11 RCTs	PLWD & BPSD	In 2008 data supporting aromatherapy for BPSD was scarce. Studies found positive and negative consequences of aromatherapy. Side effects have not been explored.

Padilla (2011)	To examine the evidence for the efficacy of environment-based interventions on emational, behavioural and functional aspects of the lives of PLWD.	Environment	9	PLWD	Ambient music, aromatherapy, and Snoezelen are modestly effective in reducing agitation (no consistently have long-term effects).<br>Visual illusion of barriers reduces wandering but not the urge to wander. Bright light therapy can aid in regulating mood and the sleep patterns - results are preliminary. Montessori-based programmes can match activities to a person's remaining skills.
Raglio 2014	Describes the different types of evidence-based music interventions that can be found in literature and proposes a structured intervention model<br>(global music approach to persons with dementia, GMA-D).	Music	Not clear	PLWD	Does not look at the effectiveness of MT - describes the theories behind it and possible benefits
Randall 2015	Identify interventions that have the potential to reduce the occurrences of disruptive vocalisation in people affected by dementia	Music, Touch/massage	8 studies	PLWD	The overall conclusion is that the evidence base is insufficient to make recommendations for practice. However, the studies gave some indication of how research and practice might develop in this area. In particular, five elements were identified that appear to promote the best patient outcomes. These include making sure interventions are:<br>1. person-centred<br>2. individualised, adaptable<br>3. with the use of multiple approaches<br>4. carried out by staff trained in the identification of disruptive vocalisation and ways to avoid triggering these behaviours

Wall 2010	Explore how music therapy influences the behaviour of older people with dementia.	Music	13 studies	PLWD	3 themes consistently emerged: (1) music therapy reducing agitated behaviours; (2) carers having a significant role to play in the use of music therapy ; (3) music therapy resulting in positive increases in mood and socialization skills (These positive effects also extend to caregivers).
Wu 2017	To systematically evaluate the effectiveness of massage and touch on the behavioural and psychological symptoms of older people with dementia.	Touch/massage	11 studies	PLWD	Behavioural and psychological problems with older people with dementia and subgroup scores of physical aggressive behaviour, physical non-aggressive behaviour, verbal aggressive behaviour and verbal non-aggressive behaviour decreased significantly after receiving massage or touch, while the subgroups scores of anxiety, sadness and anger did not decreased significantly.
